# Triplet–triplet annihilation mediated photon upconversion solar energy systems

**DOI:** 10.1039/d3qm00069a

**Published:** 2023-04-03

**Authors:** Lukas Naimovičius, Pankaj Bharmoria, Kasper Moth-Poulsen

**Affiliations:** a The Institute of Materials Science of Barcelona, ICMAB-CSIC Bellaterra 08193 Barcelona Spain; b Catalan Institution for Research & Advanced Studies, ICREA Pg. Lluís Companys 23 08010 Barcelona Spain; c Department of Chemical Engineering, Universitat Politècnica de Catalunya, EEBE Eduard Maristany 10–14 08019 Barcelona Spain kasper.moth-poulsen@upc.edu; d Department of Chemistry and Chemical Engineering, Chalmers University of Technology Kemivagen 4 Gothenburg 412 96 Sweden; e Institute of Photonics and Nanotechnology, Vilnius University Saulėtekio av. 3 LT-10257 Vilnius Lithuania

## Abstract

Solar energy harvesting is among the best solutions for a global transition toward carbon-neutral energy technologies. The existing solar energy harvesting technologies like photovoltaics (PV) and emerging molecular concepts such as solar fuels and molecular solar thermal energy storage (MOST) are rapidly developing. However, to realize their full potential, fundamental solar energy loss channels like photon transmission, recombination, and thermalization need to be addressed. Triplet–triplet annihilation mediated photon upconversion (TTA-UC) is emerging as a way to overcome losses due to the transmission of photons below the PV/chromophore band gap. However, there are several challenges related to the integration of efficient solid-state TTA-UC systems into efficient devices such as: wide band absorption, materials sustainability, and device architecture. In this article, we review existing work, identify and discuss challenges as well as present our perspective toward possible future directions.

## Abstract 2

Triplet–triplet annihilation mediated solar energy harvesting systems for a global transition toward carbon-neutral energy technologies.

## Graphical abstract

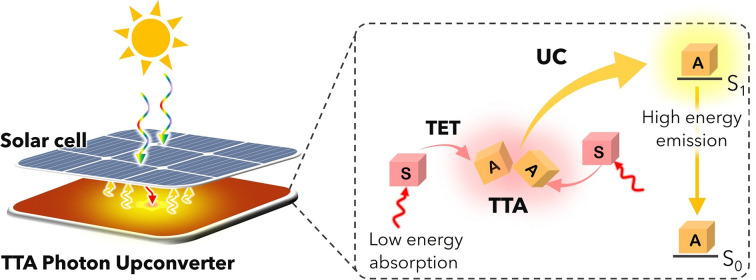

## Introduction

The solar energy of the sun has been an external power source to earth for 4.5 billion years. Around 3.4 billion years ago, the earth developed a method to store solar energy in chemical bonds through photosynthesis by cyanobacteria and later by plants.^1^ Since the discovery of fire, humans have been using various forms of stored solar energy like fossil fuels or wood to sustain life.^2–4^ However, the rise in labour-intensive industrialization of human society has exponentially increased fossil energy consumption,^5^ eventually causing global warming due to carbon emission being higher than the natural carbon fixation cycle. Further warming of the earth can pose a danger to the survival of various species including humans in the imminent future. Hence, global efforts are increasing for development of sustainable clean energy technologies with a minimum carbon footprint. Carbon-neutral energy technology is also the key agenda of the Paris Agreement,^6^ 2016 on climate change.

Solar energy technologies have emerged as a sustainable alternative to fossil energy albeit with efficiency in the range of 15–20% for commercial crystalline silicon (c-Si) solar cells.^7^ The maximal possible efficiency of single junction solar cell is described by the Shockley–Queisser (SQ) limit (33% theoretical maximum) arising due to different loss channels such as charge recombination, transmission loss of sub-bandgap photon, and thermalization loss of photons higher than the solar cell band gap.^8^ Alternatives to c-Si solar cells^9^ such as dye-sensitized solar cells,^10,11^ multijunction solar cells,^12^ and perovskite solar cells^13^ are emerging fast but still far from commercial utility due to many factors including conventional band gap related loss channels. The transmission loss (33%) can be addressed by integrating a suitable photon upconverter into the solar cell to convert transmitted photons into the solar cell absorption window.^14^ In this regard, triplet–triplet annihilation mediated photon upconversion (TTA-UC) is emerging as a promising photon upconversion process wherein two low-energy triplet excitons combine to form one high-energy singlet photon that can be infused into solar cells to overcome transmission losses.^15,16^ Hence, TTA-UC has the potential to push the solar energy conversion limit to 44%.^15^ Similar to the PV systems, solar fuel systems in general and molecular solar thermal energy storage (MOST) systems in particular also face transmission losses due to the small absorption window of molecular systems used. Practically fabricable and efficient MOST windows and chips could turn out to be a sustainable energy resource for the heating/cooling of houses and thermoelectric generators.^17,18^ Therefore, integration with TTA-UC materials can enhance the efficiency of the MOST systems by harvesting a larger amount of solar photons.

In this regard, here we have reviewed existing studies on TTA-UC integrated solar energy systems (PV & MOST) and challenges associated with the realization of efficiency enhancement. Specifically, we focus on challenges related to the fabrication of efficient solid-state TTA-UC systems with wide band upconversion, materials sustainability, PV, and MOST systems with a suitable bandgap. Elaborating, for example, (1) lack of solar thermal energy systems and TTA-UC systems with optimal spectral overlap, (2) efficiency of both the systems in the solid/semi-solid state, (3) physical losses due to scattering of the upconverted photons in all directions. We have also presented our perspective for further possible TTA-UC-PV and TTA-UC-MOST device design improvements.

However, these improvements are subject to the efficiency of TTA-UC near the solar irradiance which depends on many factors discussed below.

## Triplet–triplet annihilation photon upconversion

**Fig. 1:**
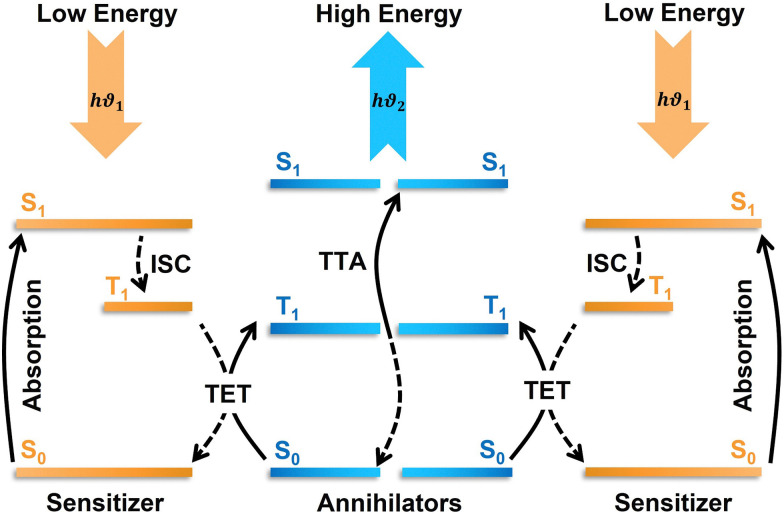
The scheme of TTA-UC with indicated energy transfer processes. ISC – intersystem crossing, TET – triplet energy transfer, TTA – triplet–triplet annihilation.

TTA-UC is a photophysical process of generating one high-energy singlet photon upon annihilation of two lower-energy triplet excitons.^19–21^ TTA-UC occurs through a series of triplet energy transfer events in an ensemble of annihilator chromophores doped with triplet sensitizer.^22^ The triplet sensitizer or donor (D) upon excitation at lower energy move to the photo-emissive triplet state either *via* intersystem crossing (ISC) through S_1_ or direct S_0_–T absorption.^19–23^ While relaxing to the ground state, the sensitizer excites the annihilator to its triplet state *via* triplet energy transfer through electron exchange. When two sensitized annihilator triplets encounter, they undergo TTA that results in the formation of a higher energy singlet state through spin inversion and emit the upconverted photon (Fig. 1).^19–26^ However, the efficacy of obtaining the upconverted photon is subject to many factors like distance, energetics, and specific orientation of the involved molecules. For example, the electron exchange between sensitizer–annihilator or annihilator–annihilator occurs *via* Dexter energy transfer (DET).^27^

The rate of DET (*k*_DET_) is inversely proportional to the sum of van der Waals radii (*L*) of the sensitizer and annihilator following eqn (1).^27^1
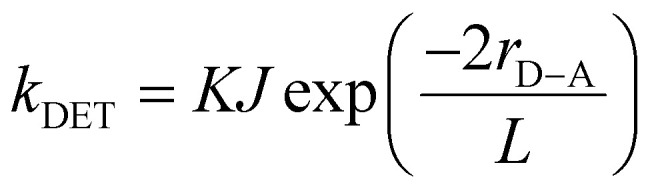
where *J* is the normalized spectral overlap integral, *K* is an experimental factor that indicates specific orbital overlap corresponding to the instantaneous orientations of a sensitizer and annihilator, and *r*_D–A_ is the distance between the sensitizer and annihilator with respect to the *L*. For DET, *r*_D–A_ should be less than 10 Å since it requires an overlap of the wavefunctions of the highest occupied or lowest unoccupied molecular orbitals (HOMO or LUMO) of the involved molecules. The normalized spectral overlap integral (*J*) can be defined by eqn (2).2
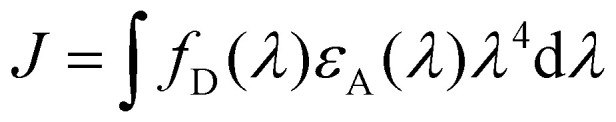
where (*f*_D_) and (*ε*_A_) are normalized emission intensity of a donor and normalized extinction coefficient of an annihilator, respectively.

The performance of TTA-UC is evaluated using upconversion quantum yield (*ϕ*_UC_) that is a function of many other factors related to eqn (3).3
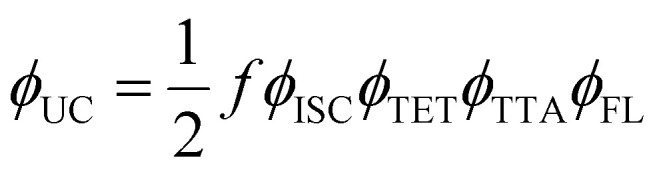
where *ϕ*_ISC_, *ϕ*_TET_, *ϕ*_TTA_, *ϕ*_FL_ denote quantum yields of the intersystem crossing in the sensitizer (ISC), sensitizer to annihilator triplet energy transfer (TET), triplet–triplet annihilation (TTA), and fluorescence of the annihilator (FL). The factor *f* denotes the statistical probability of the formation of one emissive singlet state upon annihilation of two triplet excitons, and ½ indicates the emission of one photon upon absorption of two photons, which sets the limit of *ϕ*_UC_ to be 50%.

Experimentally, *ϕ*_UC_ is calculated *via* relative method using reference standard^28,29^ or absolute method using integrating sphere.^30^ In the relative method, a reference fluorescent dye or upconversion system having an overlapping absorption with the triplet sensitizer of the TTA-UC system under investigation is used as a reference standard using eqn (4).^28,29^4
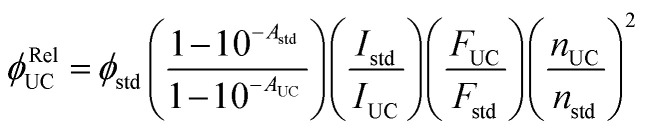
where *ϕ*_UC_, *ϕ*_std_, *A*_UC_, *A*_std_, *I*_UC_, *I*_std_, *F*_UC_, *F*_std_ and *n*_UC_, *n*_std_ are quantum yield, absorbance, excitation intensity, integrated UC emission profiles, and refractive index of the UC sample and reference standard, respectively. While the relative method remains suitable for *ϕ*_UC_ calculation in the solution state, the integrating sphere is preferred for solid-state measurements to avoid thickness-related absorption errors. However, inherent self-absorption of the UC photons and reabsorption of the emitted UC photons reflected by the integrating sphere are secondary loss channels that give errors in the absolute *ϕ*_UC_ measured in the integrating sphere. Kimizuka and Yanai's group^30^ devised a method to address the reabsorption of the reflected UC photons. For this, they calculated the reabsorption probability (*a*) in the integrating sphere to correct absolute 
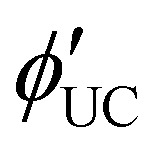
 calculation according to eqn (5).^30^5
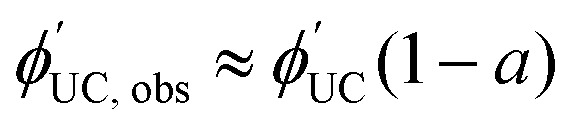
where 
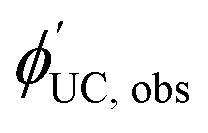
 is observed UC quantum yield and 
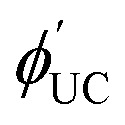
 is the UC quantum yield corrected for reabsorption probability. The *a* is evaluated by eqn (6).6
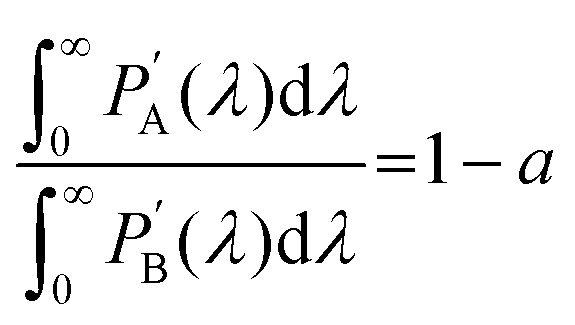
where, 
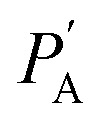
 and 
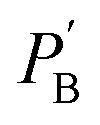
 are UC photoluminescence spectra in photons per wavelength normalized with integrated phosphorescence spectra of a sensitizer, measured inside and outside of the integrating sphere. The UC quantum yields calculated using this method give similar values to that calculated using a relative method in solution.^30^ It was also found true for green to blue bioplastics film^31^ thus validating its applicability towards solid-state TTA-UC systems.

The inherent UC photon losses due to the secondary inner filter effect, caused by the reabsorption of UC photons by the sensitizer due to overlapping spectra can be corrected by the conversion factor (*F*_c_) as per eqn (7).^32^7
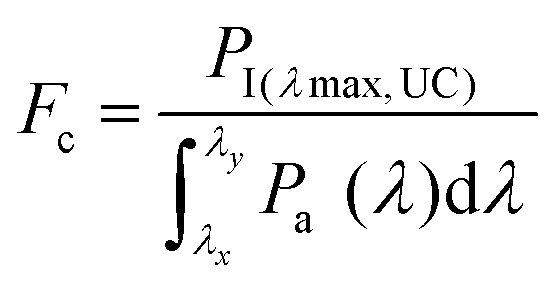
where *P*_I_ and *P*_a_ are normalized photoluminescence intensity at maximum UC emission wavelength of annihilator and normalized integrated emission of annihilator at low concentration, respectively. Consequently, the correct *ϕ*_UC_ can be calculated using eqn (8).^32^8
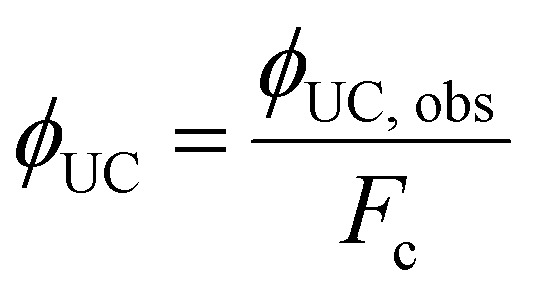
However, it must be noted that only *ϕ*_UC,obs_ will be used upon TTA-UC integration with the devices. The only way to avoid inherent reabsorption losses is a suitable design of the sensitizer–annihilator pairs with non-overlapping spectra and fabrication of low-thickness samples.

**Fig. 2:**
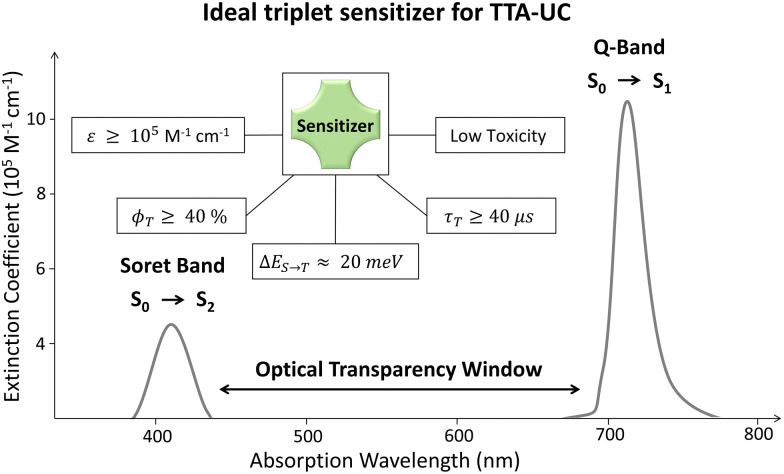
Illustration of the photophysical properties of an ideal triplet sensitizer for TTA-UC.

The quantum yield of each energy transfer step within the TTA-UC systems is of significant importance to obtain maximum *ϕ*_UC_ required for efficient integration with solar energy systems. Therefore, we will briefly discuss the state-of-art to obtain a complete picture of the energetic requirements for sensitizer, and annihilator molecules and the progress made to achieve these requirements. Basically, the quantum yield (*ϕ*) is the number of photons emitted per number of photons absorbed by the system.^33^ Therefore, when considering device integration of TTA-UC, the sensitizer being a key absorber must possess the following properties (Fig. 2). First, it must have a broad absorption cross-section in addition to a high molar extinction coefficient (*ε*) to harvest maximum photons. Second, the sensitizer must have a small energy gap between the singlet and triplet states (Δ*E*_S–T_) for a high rate of spin-forbidden ISC (*k*_ISC_). Third, because of the bimolecular nature of TTA-UC, the sensitizer must have a long triplet lifetime to meet an annihilator for transferring energy in its lifetime. And fourth, it must have a broad optical transparency window between the bands preceding the key absorption band to avoid inherent reabsorption of the upconverted photons by the sensitizer. For example, the region between the Q-band (S_0_–S_1_) and Soret band (S_0_–S_2_) of the porphyrins.^34^

Most of the triplet sensitizers used in TTA-UC are organometallic complexes (porphyrins, phthalocyanines, or their metal–organic) complexes,^35–38^ thermally activated delayed fluorescence (TADF)^39,40^ molecules, and BODIPY dyes^35,41^ which all have their strengths and challenges. For example, the red/green light-absorbing transition metal porphyrins have a reasonable phosphorescence quantum yield (*ϕ*_T_ = 5% to 50%) and phosphorescence lifetime (*τ*_T_ = 20 μs to 300 μs) but low *ε* of the Q-band as compared to the Soret band. Metal phthalocyanines on the other hand have a significant *ε* = 2 × 10^5^ M^−1^ cm^−1^ of the Q-band and high *ϕ*_T_ > 40% but low *τ*_T_ < 5 μs.^37^ The direct S_0_–T absorbing substituted Os-complexes have emerged as a potential NIR sensitizers with zero energy loss during ISC and have *τ*_T_ = 81 μs, but have low *ϕ*_T_ = 3.1 to 5.4%, high absorption between the Soret and Q-band and a small Stokes shift.^35,41^ Due to the toxicity of transition metals, halogenated BODIPY dyes with high *ε* and *ϕ*_T_ have emerged as alternative photosensitizers.^35,41^ The introduction of heavy atoms increases the *ϕ*_ISC_ of BODIPY due to the increased spin–orbit coupling that decreases the Δ*E*_S–T_ to increase the *ϕ*_T_.^41^ However, the application of BODIPY in TTA-UC is mainly limited to Vis-Vis TTA-UC and realisation of NIR absorbing BODIPY is needed for practical application in solar energy storage systems. The TADF or multi-resonance TADF (m-TADF) are emerging as another class of promising sensitizers due to small Δ*E*_S–T_ (20–40 meV), but the rate of reverse intersystem crossing (RISC) is a limiting step that should be outperformed for high *k*_ISC_. However, the TADF have low *ε* and small optical transparency window that are essential for solar energy harvesting applications. Apart from the organic/metal organic sensitizers, the quantum dots^42–44^ and metal halide perovskite nanocrystals^45^ have emerged as promising triplet sensitizers due to high *ε* and tunable absorption/emission. However, they have many challenges for practical applications such as complicated synthesis, high excitation intensities (W cm^−2^), the strong back Förster resonance energy transfer (FRET), *etc.* that affects the triplet energy transfer to annihilator. Hence, an ideal TTA-UC sensitizer must have a low toxicity, *ε* ≥ 10^5^ M^−1^ cm^−1^ of Q-band, broad optical transparency window between Soret band and Q-band (for porphyrins and phthalocyanines), *ϕ*_T_ ≥ 40%, Δ*E*_S–T_ ≈ 20 meV, and *τ*_T_ ≥ 30 μs. Additionally, the sensitizer must have an appropriate molecular orientation (factor *K* in eqn (1)) or suitable transmitter/mediator for high *ϕ*_TET_ to the annihilator.

The *ϕ*_TET_ between the sensitizer and annihilator molecules is usually regarded as a challenging step in TTA-UC systems. The TET between the molecules is accomplished *via* DET, that has a distance limit of up to 1 nm and functions according to eqn (1). In the solution state, due to the high molecular diffusion the *ϕ*_TET_ can be enhanced by increasing the annihilator concentration to maximize the probability of sensitizer–annihilator collision. However, the aggregation of polyaromatic dyes at high concentrations can cause emission quenching in addition to primary and secondary inner filter effects.^36,46,47^

Dimeric and polymeric annihilators of DPA/perylene have emerged as alternatives to limit the concentration factor.^48,49^ In such systems, triplet energy can migrate intramolecularly in the dimers or polymers to minimize the energy loss due to diffusion and to increase the TTA rates, but have typically been limited to Vis to Vis photon upconversion. Additionally, they face challenges with aggregation quenching in the solid-state which is key for practical applications of TTA-UC. Recently, Congrave *et al.* found a synthetic route to suppress aggregation-induced fluorescence quenching with a conjugated polymer-strapped derivative of diphenylanthracene (DPA polymer).^50^ Interestingly the DPA polymer showed a similar fluorescence quantum yield (*ϕ*_f_ ≈ 40%) both in solution and spin coated solid-state films. Thus gave new synthetic directions for an annihilator design for solid-state devices.

The *ϕ*_TET_ can also be increased by attaching a triplet energy transmitter/mediator to the sensitizer. For example, the case with a quantum dot or perovskite nanocrystal-based sensitizers. However, these systems have been also found efficient only in the solution state due to the phase separation of quantum dots in the solid-state.^51^ Another promising way to increase the *ϕ*_TET_ is a suitable tuning of the annihilator molecular structure so that it has triplet energy levels very close to that of the sensitizers (Δ*E*_T_ ≥ 4*k*_b_*T*).^38,52^

**Fig. 3:**
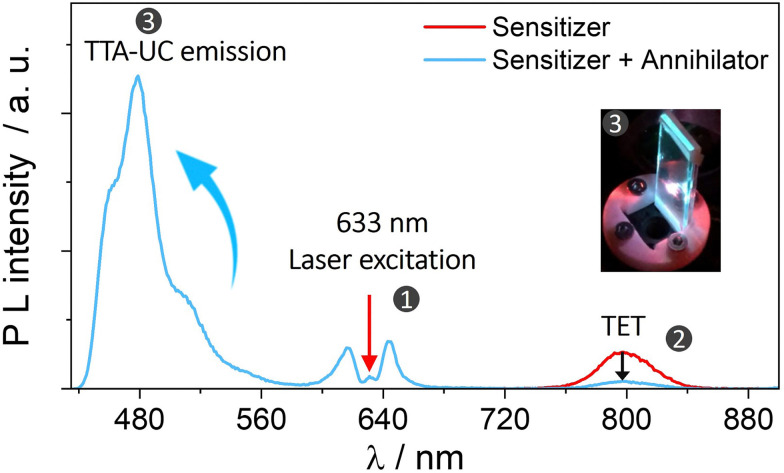
Plot illustrating UC emission of annihilator (blue line) at the expense of sensitizer's phosphorescence (red line) due to subsequent TET and TTA processes in the solid-state bioplastic film (inset). Part of the figure is reproduced from ref. 32 with permission from the Royal Society of Chemistry.

The *ϕ*_TET_ is generally calculated from quenching of the phosphorescence quantum yield (*ϕ*_p_) or lifetime (*τ*_p_) of sensitizer with and without (*ϕ*_po_, *τ*_po_) annihilator according to eqn (9).9
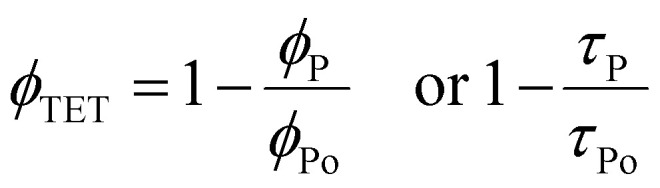
An example of TTA-UC in the solid-state demonstrating an increase in emission of annihilator at the expense of sensitizer's phosphorescence due to TET and subsequent TTA is shown in Fig. 3.

Other than *ϕ*_UC_, threshold excitation intensity (*I*_th_) of the TTA-UC system is an important parameter for solar energy applications. An efficient system with *I*_th_ near the solar irradiance would be more suitable for practical application. Therefore in the coming section, we will discuss *I*_th_ concerning solar energy applications.

### Upconversion threshold

TTA-UC is a process that depends on the excitation power density in two stages. The first stage is a quadratic dependence of UC emission intensity on excitation power density which marks the non-saturation regime as the concentration of triplet excited molecules is relatively low. In this case, the TTA process may appear rarely as the collision of two excited molecules is required to produce an emissive singlet state. That is why the probability of TTA grows quadratically with excitation power density until almost half of the molecules are excited. The second stage of TTA-UC is a linear growth of UC emission with excitation power density. This regime shows a saturation concentration situation of excited molecules where each additional triplet excited state finds an already existing counterpart to undergo TTA. As it follows, the intersection of quadratic and linear regimes is called the UC threshold (*I*_th_) and equals 38.2%^53^ of a maximum UC emission intensity or quantum yield. The *I*_th_ can be defined as follows:^54^10
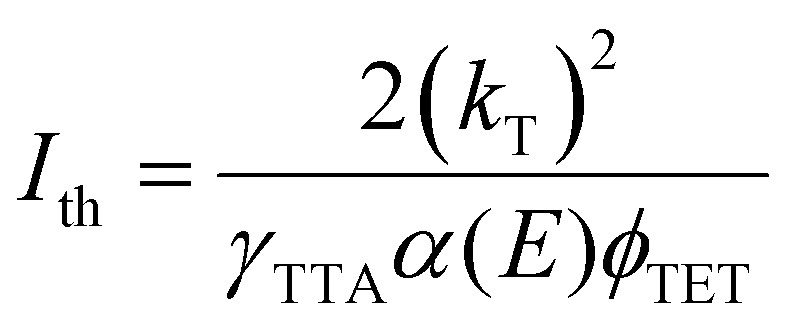
where *k*_T_ is the triplet decay rate, *γ*_TTA_ is bimolecular annihilation constant, *α*(*E*) is sensitizer's absorption at excitation energy, *ϕ*_TET_ is TET quantum yield.

Usually, the *I*_th_ is required to be much lower than the solar energy density of a certain region for TTA-UC to operate efficiently. The *I*_th_ was previously thought to be equal to 50% of the maximum UC quantum yield (*ϕ*^∞^_UC_), however, recent work by Murakami *et al.*^53^ clarified that this point corresponds only to 38.2% of the *ϕ*^∞^_UC_. Also, authors highlighted a new way to determine the *I*_th_ and *ϕ*^∞^_UC_ accurately by fitting the experimental dependence of *ϕ*_UC_ on excitation intensity (*I*_ex_) even if the saturation regime is not achieved as per eqn (11).11
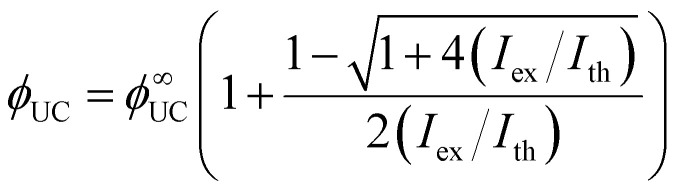
This, in turn, allows evaluation of the statistical probability factor (*f*) of the annihilator by using *ϕ*^∞^_UC_ of the system as the *ϕ*_TTA_ approaches unity, which we will discuss in the next section.

### Statistical probability factor

The statistical probability factor (*f*) is an intrinsic property of an annihilator molecule that refers to the probability of a singlet state generation through the annihilation of two triplets. The annihilation takes place due to the coupling of the wavefunction of two triplet spin states aligned either parallel or antiparallel to each other. It can be simply expressed in Heisenberg's spin only Hamiltonian, eqn (12).^55,56^

**Fig. 4:**
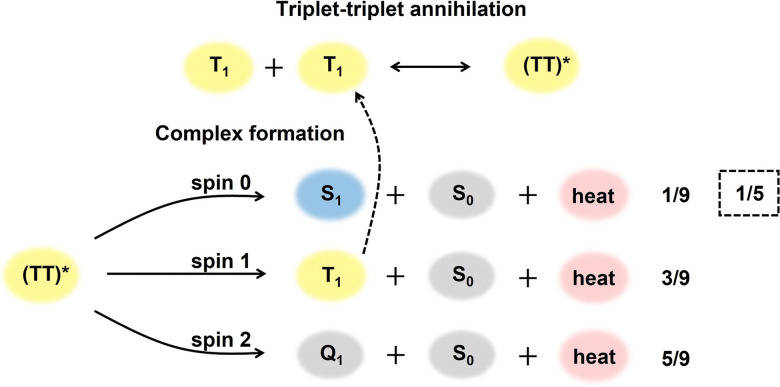
The scheme of a singlet, triplet, and quintet encounter complex formation *via* TTA. The statistical probabilities for each encounter are provided. The dashed line represents the triplet state recycling process increasing *f* to 1/5.

12 *Ĥ* = −2*JŜ*_1_·*Ŝ*_2_

where *Ŝ*_1_ and *Ŝ*_2_ are individual spin operators of the interacting triplets and *J* is the magnetic exchange parameter. The *J* contains all of the spatial information of the wave function for coupling through-space and through-bond interactions which determine the ground-state spin preferences. The triplet coupling results in 9 spin-pair eigen states; 1 singlet, 3 triplets, and 5 quintets which are graded corresponding to the spin multiplicities in the ratio of 1 : 3 : 5 according to the Glebsch–Gordan series, respectively (Fig. 4).^57^ As specified by the spin statistics, the limit of the *f* is defined as 1/9, but the possibility to recycle a triplet state from quintet or higher energy triplet can enlarge this theoretical value to 1/5.^58^

However, this is possible only if the coupling of triplets occurs *via* anti-ferromagnetic coupling (AFC), which reverses the energetic order of spin states against the Hund's rule according to eqn (13).^55^

**Fig. 5:**
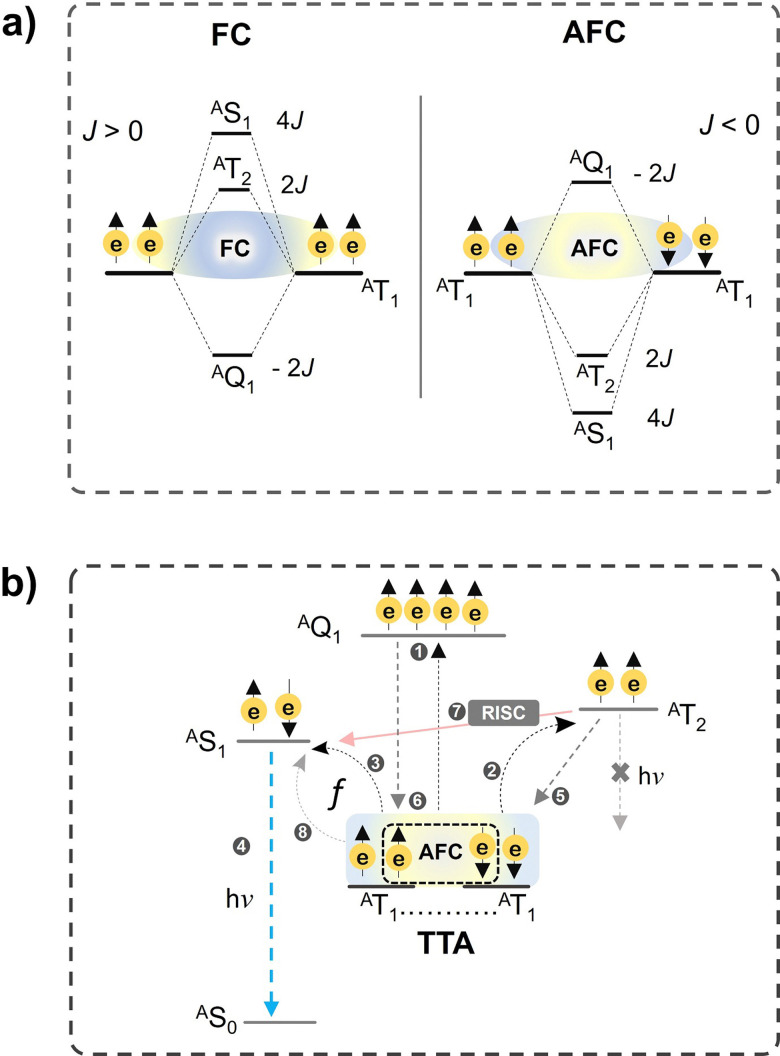
Illustration of (a) ferromagnetic (FC) and anti-ferromagnetic coupling (AFC) of triplets, and (b) post-TTA events like UC emission and reverse intersystem crossing (RISC), and quintet or triplet recycling to the T_1_. (b) is reproduced from ref. 21 with permission from the Royal Society of Chemistry.

13 *E*_s_ = −*J*(*S*(*S* + 1) − 4)

where *E*_s_ is the energy of the spin-state, *J* is the magnetic exchange parameter and *S* is the total angular momentum of the annihilation triplets. If *J* > 0, similar spins of two triplets interact *via* ferromagnetic coupling (FC) making the quintet the lowest energy state, and if *J* < 0 opposite spins of two triplets interact *via* anti-ferromagnetic coupling (AFC), hence, the quintet becomes the highest energy state followed by triplet and singlet (Fig. 5a). It can be understood by putting the value of *J* = +1 or −1 and *S* = 0, 1 and 2 for singlet, triplet and quintet state in eqn (13). As it can be seen from Fig. 5b, that post-TTA events discards complete quenching of the photons of higher energy quintet and triplet states. Hence they can participate in TTA coupling to increase the spin statistical probability of singlet formation beyond 1/9 *via* reverse intersystem crossing (RISC)^59^ or spin mixing of singlet–quintet states.^60^

Experimentally, *f* can be defined according to eqn (14) where it depends on *ϕ*^∞^_UC_ and energy transfer processes within the TTA-UC system.^36,37,61^ Also, the *f* value depends on bimolecular annihilation constant (*γ*_TTA_), annihilation radius (*R*) and diffusion coefficient (*D*) of annihilator as described by Köhler *et al.*^57^ in eqn (15). Therefore, *f* depends on the molecular structure, energy state distribution, and orientation of the annihilator molecule during triplet coupling.14
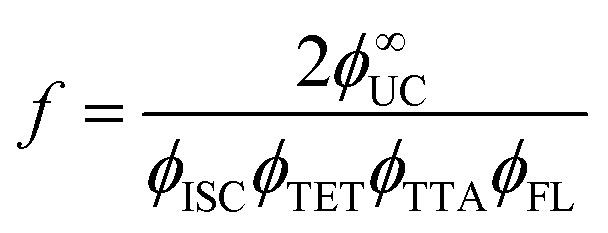
15
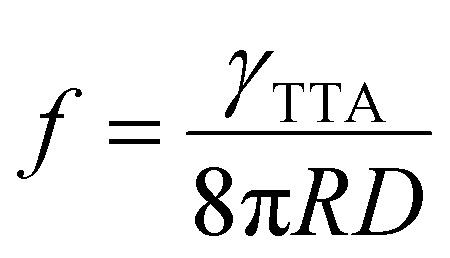
For example, the *f* of some annihilators (DPA, TIPS-naphthalene, *etc.*) have been reported to exceed 1/5^36,49,62,63^ as their energy state distribution is suitable for the TTA process (2T_1_ > S_1_) and hinders other non-radiative decay channels such as singlet-fission (SF) or high energy triplet states (T_*n*_). The 2T_1_ energy loss to T_*n*_ was theoretically predicted to be the dominating non-radiative decay channel negatively affecting the *f*.^58,64^ Recently, it was proven with diketopyrrolopyrrole (DPP) emitters that decreasing the energy gap between 2T_1_ and T_2_ was responsible for the decrease in *f*.^61^ Hence it is essential to have a large energy gap between 2T1 and T_*n*_ energy to have high *f*.

**Fig. 6:**
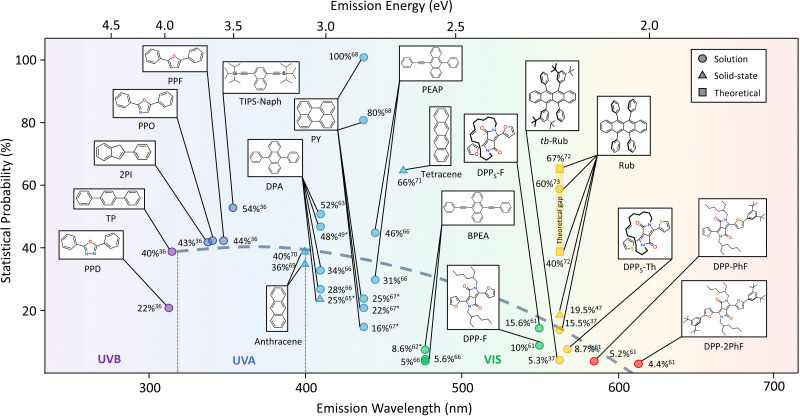
Plot showing variations in statistical probability factor values for TTA annihilators over UVB-to-red spectral range in solution (circle), solid-state (triangle). The values in square show value calculated theoretically. The dashed line indicates variation of statistical probability factor with an increasing emission wavelength of annihilators. Values marked with asterisk (*) are not directly reported but deduced from the data presented in publications.

Considering the possible importance of *f* in annihilator design to achieve desired UC quantum yield, we have reviewed the reported *f* values of the widely used TTA annihilators over the UV-Vis spectral range to find possible trends or structure–property relations (Fig. 6). The *f* values for some of the annihilators have been calculated by us using the method proposed by Murakami *et al.*^53^ from the published data of other parameters considering *ϕ*_TTA_ = 1, when *ϕ*^∞^_UC_ becomes invariant with excitation intensity and has been shown as approximate value.

Interestingly, barring a few exceptions, we found a trend of decrease in *f* values of TTA annihilators with an increase in branched conjugation from UV to NIR region in solution. For example, an average *f* ≈ 40% was observed for the UV-B (PPT, TP)^36^ and UV-A (2PI, PPO, PPF)^36^ region that decreased to *f* ≈ 25 to 35% in the blue region (DPA, perylene, PEAP),^49,63,65–67^*f* ≈ 10 to 15% in the green region (BPEA, DPPs-F, DPP-F),^61,62,66^*f* ≈ 10 to 20% in the yellow region (rubrene, tb-rubrene, DPPs-Th),^37,47,61^ and *f* ≈ 5% in the red-region (DPP-PhF, DPP-2PhF).^61^ An exceptionally high *f* ≈ 54%^36^ was observed for TIPS-Naph showing high *ϕ*_UC_ = 17% highlighting the importance of *f* in the final output of TTA in addition to other energy transfer steps. It was also found true for DPA^49,63,65,66^ and perylene,^46,67,68^ although with a lot of variations in *f* observed by different research groups (Fig. 6). The *f* value of perylene in particular is a bit disputed since there is a large variation from 16%^67^ to 100%.^68^ For example, *f* = 100%^68^ seems to be overestimated as the T_2_ state in perylene tends to be around 3.1 eV^46,67^ and not at 4.0 eV as reported.^68^ This means that T_2_ is not energetically far from 2T_1_, hence there is an accessible non-radiative loss channel to alter *f*. Interestingly, *f* increased with an increase in linear conjugation from anthracene (*f* = 36–40%)^69,70^ to tetracene (*f* = 66%)^71^ in crystalline state. But there are not any available reported crystalline systems with enhanced linear conjugation to find any trend. Rubrene has been the leading yellow emitting annihilator in TTA-UC. Recenlty, Bossanyi *et al.*^72^ modelled *f* values of rubrene in different molecular orientations and showed that *f* changes from 40% to 67% from perpendicular to parallel orientation due to spin-mixing of spin-pair wavefunctions.^72^ The values are close to the experimental value (*f* = 60%), reported by Schmidt *et al.*^73^ But that experiment was carried out using high energy pulsed laser.^73^ Moreover, the maximum *f* = 15.5% (solution) and 19.5% (solid-state) has been reported for native rubrene^37,47^ that decreases further to 5.3% due to *tert*-butylation in steady-state measurements.^37^ Hence, the lower *f* could be one of the main reasons for low TTA-UC quantum yield observed for rubrene based TTA-UC systems.^37,47^ However, we strongly believe that molecular orientation during inter-triplet coupling plays a key role in deciding the *f* value^72,74^ and side chain branching or enhanced conjugation alters most suitable orientation for effective coupling. It was also found true for red-emitting annihilators showing low *f* values of around 5%.^61^

The observed trend may also be influenced by the energy gap law^75,76^ considering the differences in achievable higher triplet states (T_*n*_). For example, in the UV-blue region, the 2T_1_ is able to access the T_*n*_ states leading to higher *f* values possibly due to the higher T_*n*_–T_1_ and T_1_–S_0_ energy differences compared to the lower energy emission annihilators. The higher T_*n*_–T_1_ energy differences may impede the internal conversion to T_1_, therefore leading to more pronounced RISC to S_1_. This, in turn, might have a positive effect on *f*. However, in the green to red region, the T_*n*_–T_1_ and T_1_–S_0_ energy differences are smaller and, according to the energy gap law, may facilitate faster decay of triplets affecting the possibility to recycle the T_*n*_ states efficiently. As follows, this cause may contribute to the reduced *f* values. The trend also shows that possible far-red and NIR annihilators may not have a significant *f* value to harvest photons from below the crystalline silicon band gap to realize high UC quantum yield for practical implementations in the far-red and NIR region if suitable molecular orientation for inter-triplet coupling is not achieved.

## Solid-state TTA-UC for solar energy systems

While most efficient TTA-UC systems have been reported in degassed solutions,^77–79^ practical integration with solar energy systems requires them to function efficiently in the solid-state. But the fabrication of the efficient solid-state TTA-UC faces many challenges: (1) aggregation-induced quenching of singlet/triplet energy of chromophores; (2) triplet quenching by molecular oxygen; (3) small upconversion windows, *etc.*^22,80,81^ Additionally, the non-recyclability of petro-polymers used for solid-state fabrication and the toxicity of TTA-UC chromophores is another challenge that hampers the sustainability of solid-state TTA-UC materials.^82,83^ Many of these challenges have been addressed by employing various solid-state fabrication strategies. For example, by tuning the molecular structure of sensitizer–annihilator, their concentrations, crystallization, glassification, doping in amorphous polymers and bioplastics, *etc.*^22,80^ From the application point of view, the solid-state TTA-UC systems can be divided into three categories: (1) near-infrared to Visible (NIR-to-Vis); (2) visible to Visible (Vis-to-Vis) and; (3) visible to ultraviolet (Vis to UV). Under the current scope of this review, we will mainly discuss the most efficient solid-state systems (Table 1) as well as their applications in solar energy systems. Detailed information about the developments in solid-state TTA-UC systems can be found in the papers published elsewhere.^21,80,84^

**Table 1:** Most efficient solid-state TTA-UC systems over UV-to-NIR spectral range. Matrix, polymer glass transition temperature (*T*_g_), energy difference of anti-Stokes shift (Δ*E*_UC_), UC threshold (*I*_th_) and UC quantum yield (*ϕ*_UC_) indicated. Δ*E*_UC_ here is presented as difference between wavelength of excitation and maximum of emission wavelength

Sensitizer–annihilator-emitter	Matrix	*T*_g_ (°C)	UC range (nm)	Δ*E*_UC_ (eV)	*I*_th_ (W cm^−2^)	*ϕ*_UC_ (%)
Near infrared sensitized TTA-UC (*λ*_ex_ > 780 nm)						
Os(atpy)(tbbpy)Cl^+^-rubrene-DBP^85^	PVA film	80	938 to 625	0.66	125	2.05
PdTPTAP-rubrene^88^	PVA film	80	810 to 560	0.68	1.40	3.65
PdSNC-rubrene-DBP^86^	Amorphous film	—	808 to 610	0.50	—	0.53
Y6-rubrene^138^	Thin bilayer	—	850 to 575	0.70	—	0.52

Far-red sensitized TTA-UC (700 nm < *λ*_ex_ < 780 nm)						
MoSe_2_-rubrene-DBP^87^	Thin bilayer	—	772 to 610	0.43	0.26	1.1
PdPc-rubrene^47^	Polystyrene	100	730 to 575	0.46	1.40	1.2

Red sensitized TTA-UC (620 nm < *λ*_ex_ < 700 nm)						
PdTPBP-TIPS-anthracene^32^	Gelatin	—	633 to 478	0.64	0.095	8.8
PdTPBP-anthracene^89^	Crystals	—	635 to 470	0.69	0.017	5
Pd(OBu)_8_Pc-rubrene^90^	Polyacrylate	44–48	670 to 560	0.36	—	15

Green sensitized TTA-UC (532 nm < *λ*_ex_ < 550 nm)						
PdOEP-DPA^90^	Polyacrylate	48–52	543 to 435	0.57	0.0014	16
PtOEP-DPA^91^	Polyurethane	−42	532 to 430	0.55	0.022	11
PtOEP-DPA^139^	LAPONITE®/PVP	—	532 to 435	0.57	0.033	23.8
PtOEP-DPA^65^	PMMA	108	532 to 430	0.55	0.005	8
PtOEP-ANNP^92^	Crystals	—	542 to 434	0.57	0.0007	16
PtOEP-C7-sDPA^93^	Crystals	—	532 to 434	0.53	0.005	10

Blue to UV TTA-UC (400 nm < *λ*_ex_ < 532 nm)						
Ir(ppy-DBP)-DBP^100^	Polyurethane	−42	450 to 370	0.53	—	1.3

Most of the efficient NIR and far-red sensitized TTA-UC systems have used rubrene as a key annihilator and dibenzotetraphenylperiflanthene (DBP) as a singlet acceptor/emitter.^85–87^ Since rubrene satisfies the energetic condition for singlet fission *i.e.* S_1_(*E*) ≥ 2T_1_(*E*), the upconverted singlet photon undergo singlet fission before emission, which reduces the overall *ϕ*^∞^_UC_. DBP is used to avoid this loss channel *via* singlet energy transfer from rubrene to DBP because of its low-lying singlet state compared to rubrene (Δ*E*_S_ ≈ −0.14 eV).^43,83^ Eventually, DBP emit the upconverted photon of rubrene at low energy in the red-region. In such systems, the best *ϕ*_UC_ = 2.05% has been reported for Os(atpy)(tbbpy)Cl^+^-rubrene-DBP nanoparticles doped polyvinyl alcohol (PVA) film upon 938 nm excitation.^85^ Without DBP the best *ϕ*_UC_ = 3.65% has been reported in Pd-tetraphenyltetraanthroporphyrin (PdTPTAP)-rubrene emulsified mesoporous PVA films upon 810 nm excitation.^88^

For red light sensitized TTA-UC, thermally evaporated rubrene on palladium phthalocyanine (PdPc) doped polystyrene surface (*ϕ*_UC_ = 1.2%)^47^ and rubrene-DBP spin-coated on an atomically thin two-dimensional (2-D) layer of MoSe_2_ as sensitizer (*ϕ*_UC_ = 1.1%)^87^ were found to be the most efficient. Incidentally, most efficient solid-state TTA-UC system have been reported for Vis-to-Vis TTA-UC.^32,89–93^ It could be due to the combined effect of high statistical probabilities of the annihilators, small anti-Stokes shifts, and suitable orientations of sensitizer–annihilator pairs for TET and later triplet coupling in solid-state. For example, a high *ϕ*_UC_ = 15% in air has been reported for Pd(ii)-octa-butoxy phthalocyanine Pd(OBu)_8_Pc-rubrene nanoemulsion doped polyacrylate films upon red light (*λ*_ex_ = 670 nm) excitation.^90^ This is despite low *f* value of rubrene. In the same framework the green light (*λ*_ex_ = 543 nm) sensitized palladium octaethylporphyrin (PdOEP)-diphenylanthracene (DPA) film showed *ϕ*_UC_ = 16%.^90^ This is due to the higher molecular diffusion of the chromophores inside the liquid core of nanoemulsions that fosters high TET and effective inter-triplet coupling to populate the singlet state.

However, synthetic polymers as a matrix may not be a sustainable solution for a long period due to non-recyclability. Hence, an alternative recyclable matrix is desired to fabricate an efficient TTA-UC system. Here, recyclable TTA-UC bioplastics, also operating *via* molecular diffusion mechanism could be a viable solution. For example, TTA-UC bioplastics comprised of chromophores doped viscous surfactant liquids dispersed inside the biopolymer matrix have shown high *ϕ*_UC_ = 8.8% for red to blue,^32^ and *ϕ*_UC_ = 7.8%^31^ for green to blue UC in air. Moreover, chromophores used in these bioplastics could be recycled *via* simple solid–liquid phase separation that enhances their sustainability factor.

Another way to avoid synthetic plastics is the air-stable TTA-UC crystal, which operates *via* triplet energy migration (TEM). It can be achieved *via* a suitable molecular design of chromophores and a crystallization approach. For example, molecular engineering of anthracene to 9-(2-naphthyl)-10-[4-(1-naphthyl)phenyl]anthracene (ANNP) and subsequent crystallization with platinum octa-ethyl porphyrin (PtOEP) and annealing yielded high-end results with *ϕ*_UC_ = 16.4%, *I*_th_ = 0.7 mW cm^−2^ in air.^92^ Similarly, co-crystallization of PtOEP with cyclic diphenylanthracene derivative, C7-sDPAm yielded *ϕ*_UC_ = 10% and *I*_th_ = 5 mW cm^−2^ in air.^93^ Fabrication of crystalline mono/bilayers of such TTA-UC crystals beneath the solar cells of suitable band gap can certainly enhance their power conversion efficiency (PCE) by manifold. Moreover, such molecular designs also inspire the engineering of annihilators capable of upconverting NIR light to increase the efficient upconversion width of TTA-UC crystals. But the longevity of TTA-UC in crystals needs to be established for practical integration with solar harvesting devices.

The commercial crystalline solar cell (c-Si), has a band gap of ≈1.1 eV,^94^ that is below the longest energy-converting solid-state TTA-UC system (1.32 eV).^85^ Therefore, the current TTA-UC systems are not suitable for integration with c-Si. Instead, they are suitable for integration with high band gap solar cells, which are currently at the development stage. However, efforts have been made in the past decade, with several proof-of-concept TTA-UC integrated high band gap PV devices. Therefore, in the next section, we will review these developments concerning the sensitizer–annihilator design, TTA-UC emission band gap, PV band gap, and device design.

The solid-state Vis-to-UV TTA-UC systems are rather more relevant to increase the efficiency of solar fuels, MOST systems^95^ or solid-state photoswitches.^96^ The upconversion to UV light can counter the low spectral irradiance of AM 1.5 solar spectrum in the UV region, which can be exploited to enhance the photoconversion quantum yield of photo isomers like norbornadiene, stilbene, dihydroazulene and azobenzene, *etc.*^97,98^ Recently, there has been a great surge in research on Vis-to-UV TTA-UC systems due to the discovery of TIPS-naphthalene.^79^ However, the most efficient systems (*ϕ*_UC_ = 10% to 17%)^36,79^ are limited to degassed organic solutions that are mainly exploited for photo-catalysis applications.^99^ However, if such high efficiency Vis to UV TTA-UC could be translated to solid-state it would create opportunities in several applications. Surprisingly, there is a big void in solid-state Vis to UV TTA-UC research. And the most efficient system (*ϕ*_UC_ = 1.2%) was last reported in 2016 in the form of Ir(ppy-DBP)-2,7-di-*tert*-butylpyrene (DBP) doped polyurethane films.^100^ Therefore, in the coming section we will also discuss implications of solid-state Vis to UV TTA-UC systems and our perspective for future directions.

## TTA-UC driven photovoltaic devices

Photon upconversion can overcome the transmission losses in PV devices, but practically that is needed to happen near the solar irradiance or one sun ≈100 mW cm^−2^ of AM 1.5 solar spectrum. That is where TTA-UC which functions efficiently around solar irradiance is useful compared to other UC processes like energy transfer upconversion (ETU), excited state absorption (ESA), and photon avalanche (PA), *etc.* Most of the earliest TTA-UC systems were efficient in the deaerated organic solvents. As a result, they were tested as a proof-of-concept for photocurrent enhancement of high band gap solar cells. The summary of this is provided in Table 2.

**Table 2:** Photocurrent enhancement in UC solution integrated solar cells. UC emission bandwidth, solar cell bandgap, excitation wavelength (*λ*_ex_), excitation power density (*I*_ex_) and short circuit current density due to UC (Δ*J*_sc_). Here 1 sun of 670 nm laser ≈3.89 mW cm^−2^ and 720 nm laser ≈9.81 mW cm^−2^

Sensitizer–annihilator (UC solution)	UC emission bandwidth (eV)	Solar cell	Bandgap (eV)	*λ*_ex_ (laser) (nm)	Sun	Δ*J*_sc_ (mA cm^−2^)
PQ_4_Pd-rubrene^101^	2.25–2.06	a-Si:H	1.7–1.8	670	1	0.00042
PQ_4_PdNA-rubrene^101^	2.25–2.06	a-Si:H	1.7–1.8	720	1	0.0125
PQ_4_PdNA-rubrene^102^	2.25–2.06	a-Si:H	1.7–1.8	720	1	0.035
PQ_4_PdNA-rubrene^103^	2.25–2.06	a-Si:H	1.7–1.8	670	1	0.0144
PQ_4_PdNA-rubrene^103^	2.25–2.06	P_3_HT : ICBA	2.1 : 1.72	670	1	0.0028
PQ_4_PdNA-rubrene^104^	2.25–2.06	a-Si:H	1.7–1.8	670	1	0.0075
PQ_4_PdNA-rubrene^105^	2.25–2.06	D149/TiO_2_	2.43/3.2	670	1	0.0037
PQ_4_PdNA-rubrene-BPEA^105^	2.25–2.06	a-Si:H	1.7–1.8	670	1	0.036
PQ_4_PdNA-rubrene-BPEA^105^	2.25–2.06	D149/TiO_2_	2.43/3.2	670	1	0.068
PQ_4_PdNA-rubrene^24^	2.25–2.06	D149/TiO_2_	2.43/3.2	670	1	0.0046

**Fig. 7:**
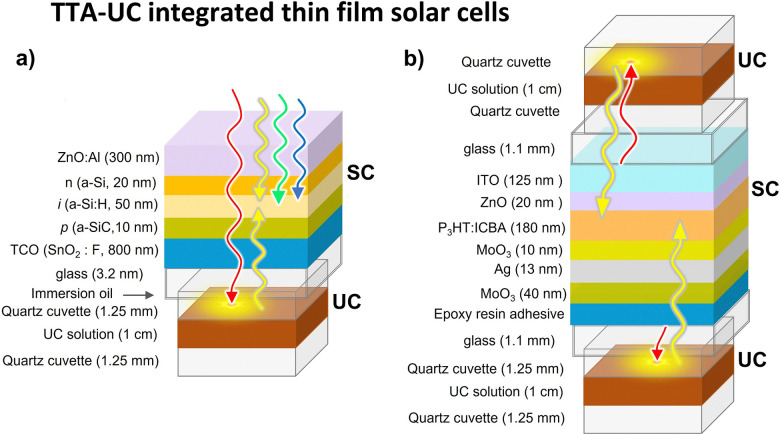
Illustration of the device structure of TTA-UC solution integrated thin film solar cell, (a) a-Si:H p–i–n solar cell and (b) P3HT:ICBA organic photovoltaic solar cell (OPV). The TTA-UC solution on both sides of OPV indicates that it can be placed either below the ITO cathode or below the Ag anode. Transmitted red light is injected back into the solar cell in the form of upconverted to yellow light.

Schmidt's research group contributed to the earliest developments of TTA-UC-PV devices with high band gap (1.7 to 3.2 eV) solar cells (a-Si:H) (p–i–n), P_3_HT:ICBA (OPV), D149/TiO_2_ (DSSC)^24,101–103^ and worked on the device design to improve the short circuit current density (*J*_sc_) and upconversion figure of merit (*ζ*). For this they mainly used TTA-UC solution of PQ_4_PdNA/rubrene, that was placed behind the solar cells to capture the back-reflected UC photons upon 670 or 720 nm laser excitation. This is because rubrene's UC emission (550–600 nm) corresponds to the band gap of the solar cells or the dye used in the case of dye-sensitized solar cells (DSSCs). The UC-enhanced short circuit current density (Δ*J*^UC^_SC_) of the solar cell was calculated using eqn (16).16
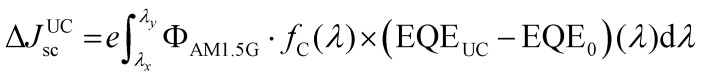
where EQE is external quantum efficiency, which is the number of charge careers extracted from the solar cell per incident photons; *e* is the elementary charge; *f*_c_ is the solar concentration factor; *ϕ*_AM1.5G_ is solar flux in photons per unit area time per wavelength. The *ζ* is calculated using eqn (17) by dividing Δ*J*^UC^_sc_ with a square of *f*_c_.17
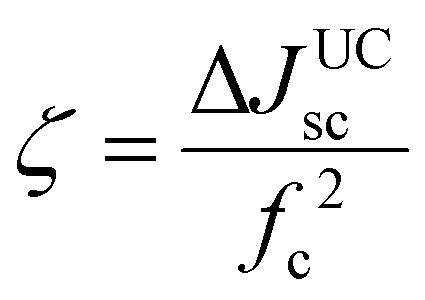
A typical device structure of TTA-UC integrated bifacial p–i–n, a-Si:H solar cell used by Schmidt's group is shown in Fig. 7a.^101^

In many papers device performance is expressed as *ζ*.^24,101–103^ But for clarity, in this paper, the current enhancement due to TTA-UC integration has been presented as change in short circuit current density only (Δ*J*_sc_). Other than changing the solar cells, they also worked on the optical design of the device to reflect a maximum number of UC photons toward the solar cell. For example, the use of silver-coated glass spheres or silver-coated PTFE as a back reflector led to a 3^102^ to 18^104^ fold enhancement in Δ*J*_sc_ (Table 2).^101,102,104^ Further, they reported around 5-fold enhancement of Δ*J*_sc_ for p–i–n, a-Si:H by changing the thickness of a-Si:H layer to 150 nm and adding annihilator, 9,10-bis(phenylethynyl)anthracene (BPEA) in PQ_4_PdNA/rubrene solution to avoid parasitic triplet loss channel (Table 2).^105^

**Fig. 8:**
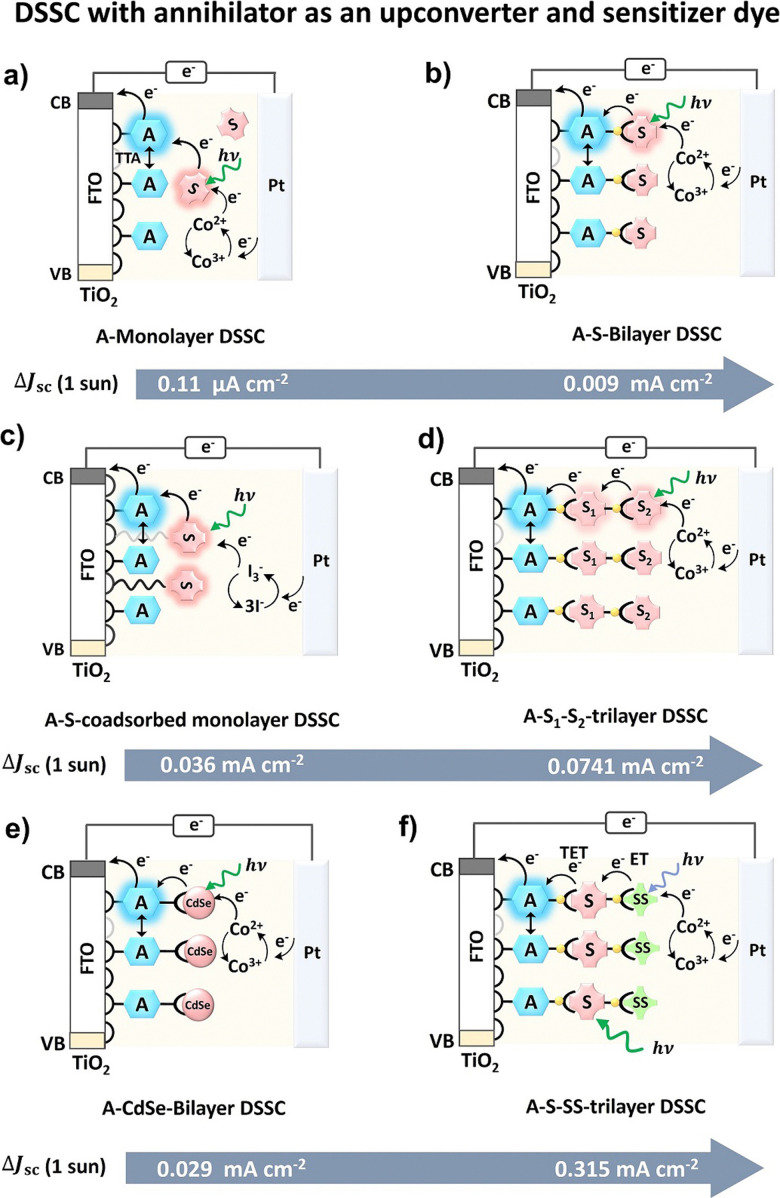
(a–f) Illustration of the development in TTA-UC integrated DSSC where an annihilator acts as an electron donor dye to the semiconductor (TiO_2_), and Co^2+^/CO^3+^ or I^3−^/I^−^ as redox mediator. A = annihilator, S = triplet sensitizer, CdSe = quantum dot sensitizer, and SS = singlet sensitizer.

The solution state TTA-UC integrated solar cells gave useful early proof-of-concept insights for PV applications of TTA-UC. However, practical PV devices require their integration in the solid-state. Moreover, they were based on the absorption of UC photons by the semiconductor or dye to increase the photocurrent. Schmidt and co-workers^106^ showed that the singlet state of anthracene could also act as an electron donor to the conduction band of semiconductor (TiO_2_) in the DSSC setup. It is due to the suitable oxidation potential of anthracene. They demonstrated a heterogenous DSSC device design where an annihilator, 4,40-(anthracene-9,10-diylbis(ethyne-2,1-diyl) dibenzoic acid) (BDCA) was adsorbed on the surface of a semiconductor (TiO_2_) and sensitizer platinum(ii) tetrabenzotetraphenylporphyrin (PtTBTPP) was dissolved in the electrolyte solution along with Co^2+^/CO^3+^ as a redox mediator. A Δ*J*_UC_ = 0.11 μA cm^−2^ of could be achieved for this device upon excitation with AM 1.5 spectrum between 560–640 nm (Fig. 8a).^106^

Hanson's research group went one step further and devised a fully solid-state TTA-UC integrated DSSC in the form of annihilator–sensitizer bilayers linked with Zn ion and fabricated on TiO_2_ or ZrO_2_ semiconductors.^107–109^ The 4,4′-(anthracene-9,10-diyl) bis(4,1-phenylene)diphosphonic acid (DPPA) and Pt(ii) tetrakis(4-carboxyphenyl)porphyrin (PtTCPP) were fabricated on to TiO_2_ or ZrO_2_ with a spatiotemporal control as anode electrode. The upconverted singlet state of DPPA injected the electron to the conduction band of TiO_2_ to start the electron flow to induce photocurrent upon absorption of the green light of solar spectrum by the PtTCPP and Co-(bpy)_3_(PF_6_)_2_ as redox mediator to this device. The resulting device showed *J*_sc_ = 0.009 ± 0.002 mA cm^−2^ upon one sun excitation of AM 1.5 solar spectrum (Fig. 8b).^108^ To further broaden the absorption range of the solar spectrum they introduced a dual sensitizer approach *i.e.* TiO_2_-DPPA-PdTCPP-PtTCPP trilayer (Fig. 8d). Due to higher photon absorption by the sensitizers, the *J*_sc_ = 0.0741 mA cm^−2^ was recorded under one sun excitation of AM 1.5 solar spectrum.^109^ Recently, they reported S_0_ to T_1_ sensitizer, carboxylated osmium(ii) polypyridyl (Os) and phosphonated bis(9,10-diphenylethynyl)anthracene annihilator (PBPEA) based DSSC to harvest NIR photons up to 950 nm. The TiO_2_-PBPEA-Zn-Os bilayered DSSC with I_3_^−^/3I^−^ as redox mediator showed Δ*J*_sc_ ∼ 3.5 μA cm^−2^ under AM 1.5 solar excitation.^110^ The low Δ*J*_sc_ despite broad absorption range was attributed to slow sensitizer to annihilator triplet energy transfer, a low injection yield for the annihilator, and fast back energy transfer from the upconverted state to the sensitizer.^110^ To avoid the sensitizer's aggregation on the annihilator-TiO_2_ film Nagata's research group co-adsorbed the sensitizer (PtTPO) on TiO_2_ with a long alkyl chain spacer (Fig. 8c). The TiO_2_-4,4′-(anthracene-9,10-diyl)bis(4,1-phenylene)dicarboxylic acid (ADDA)-PtTPO co-adsorbed film showed Δ*J*_sc_ = 0.036 mA cm^−2^ upon 1 sun excitation with simulated AM 1.5G radiation.^111^

Schmidt *et al.* reviewed these developments in 2017 and suggested Δ*J*_sc_ = 0.0741 mA cm^−2^ still short of device-relevant figure of merit *i.e.*, Δ*J*_sc_ = 0.1 mA cm^−2^ under 1 sun excitation.^112^ They suggested a roadmap to achieve the Δ*J*_sc_ ≥ 0.1 mA cm^−2^ by improving the device design, transparency of solar cell, solid-state upconverter, broadening of sensitizers absorption cross-section by new sensitization approach like semiconductors quantum dots, *etc.*

Semiconductor quantum dots are emerging as new triplet sensitizers due to their size and tunable spectrum because of the quantum confinement effect.^113^ The delocalization of their frontier molecular orbital over several nanometres results in a small singlet–triplet energy gap. Due to the long-lived triplet energy on the surface of the nanocrystals, it can be transferred to the surface-attached annihilator/acceptor *via* electron exchange. The annihilator can then transfer energy to the semiconductor of the solar cell. Hanson's research group explored this possibility and fabricated CdSe quantum dots over the TiO_2_-DPPA surface as a photoanode (Fig. 8e).^114^ The resulting DSSC showed Δ*J*_sc_ = 0.029 mA cm^−2^ upon 1 sun excitation with simulated AM 1.5G radiation. However, the obtained Δ*J*_sc_ is lower than that of DSSC with molecular sensitizer, due to the multiple factors.

For example, (1) lower sensitizer surface loadings, (2) low triplet energy transfer yield (∼40–80%), (3) slow regeneration kinetics and (4) competitive quenching of triplet excited CdSe by the Co(ii)/Co(iii)(phen)_3_ redox mediator. In an interesting development, Hanson and co-workers exploited Monguzzi's singlet sensitization approach to increase TTA-UC quantum yield to increase Δ*J*_sc_ of DSSC. The singlet sensitizer absorbs the photons in the transparency window between the sensitizer and annihilator and transfers the energy to the triplet sensitizer to increase the absorption cross-section. Fluorescein was used as a singlet sensitizer (SS) in a tri-layered TiO_2_-DPPA-PtCCP photoanode (Fig. 8f).^115^ The resulting device showed a record Δ*J*_sc_ = 0.315 mA cm^−2^ under 1 sun.

**Fig. 9:**
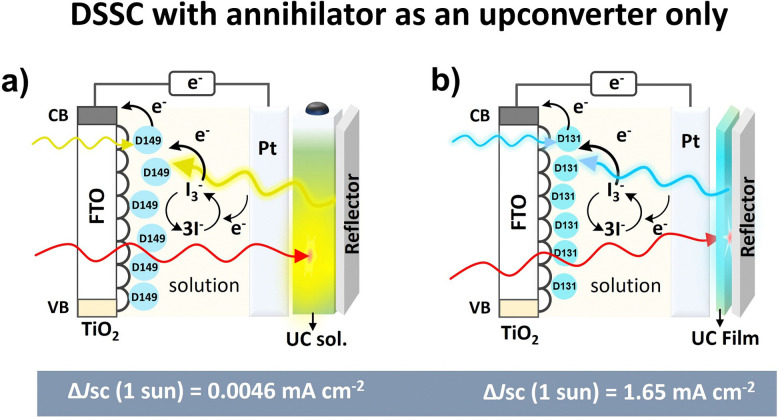
(a and b) Illustration of the development of the solution to solid-state TTA-UC integrated DSSC where UC photons of annihilator are absorbed by dye (D149 or D131) that acts as a sensitizer dye to inject an electron into the conduction band of a semiconductor.

Other than the annihilator as an electron donor, conventional DSSC with a separate electron donor dye with I^3−^/I^−^ as a redox mediator was also integrated with both TTA-UC solution and solid-state film (Fig. 9a and b).^24,116^

To harvest the maximum photon an Al_2_O_3_ reflector was placed behind the TTA-UC solution or film in these devices. The upconverted photons emitted by the annihilator were absorbed by the dye that transferred the electron to the TiO_2_ conduction band. It is due to the overlap of the absorption spectrum of dye with the emission spectrum of the annihilator. For example, the TTA-UC solution (rubrene/PQ4PdNA) integrated DSSC with D149 as an electron donor dye and rear reflector showed Δ*J*_sc_ = 0.0046 mA cm^−2^ at 1 sun excitation of 670 nm laser (Fig. 9a).^24^ They suggested poor device design for low Δ*J*_sc_. However, integration with TTA-UC film (perylene/PdTPBP doped polyurethane) with TiO_2_-D131 dye as electron donor showed enhancement of Δ*J*_sc_ = 1.65 mA cm^−2^ under 7 mW cm^−2^ (1 sun) of 635 nm laser excitation (Fig. 9b).^116^ They attributed enhancement to better device design with a background reflector, solid-state upconverter, and higher TTA-UC quantum yield. This number fits well into the device relevant Δ*J*_sc_ = 0.1 mA cm^−2^.

**Fig. 10:**
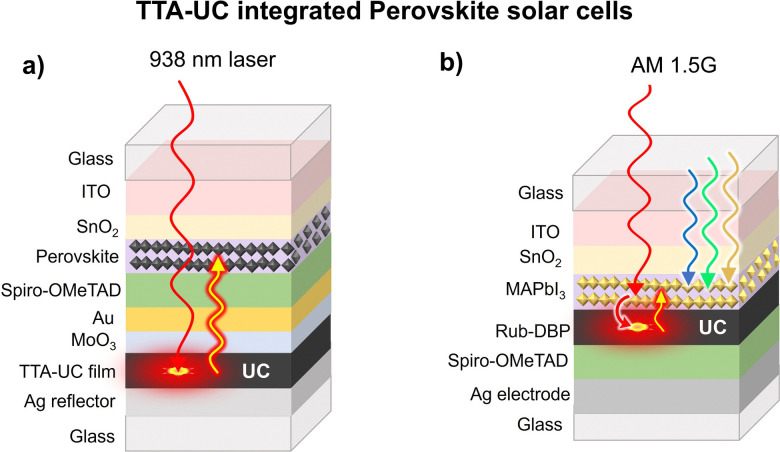
Illustration of the TTA-UC integrated perovskite solar cells. (a) Perovskites Cs_0.05_FA_0.54_MA_0.41_Pb(I_0.98_Br_0.02_)_3_ as semiconductor absorber integrated to Os(atpy)(tbbpy)Cl^+^-rubrene-DBP doped PVA TTA-UC film. (b) Perovskites, MAPbI_3_ as triplet sensitizer and semiconductor absorber crystallized on rubrene-DBP layer.

Other than thin film and DSSC, metal halide perovskite-based solar cells (PVSCs) are fast emerging as a cheap and sustainable alternative to c-Si solar cells.^117^ This is due to their exceptional properties like high extinction coefficient in the visible spectrum, the large diffusion length of charge carriers, and long excited state lifetimes.^118^ As a result, PVSCs have witnessed solar power conversion efficiency of 25% in a decade.^117,119^ However, it is still lower than the Shockley–Queisser limit.^8^ It is due to many factors, including crystal packing of perovskites, oxygen quenching, absorption limited to the visible region, *etc.* Hence, the upconversion of NIR photons by integration with TTA-UC materials can increase their efficiency. Since the maximum upconversion wavelength of TTA-UC materials is limited to 1140 nm, they are suitable for integration with PVSCs having bandgap in the visible region.^21,120^ Kimizuka, Yanai, and co-workers, integrated PVSC's Cs_0.05_FA_0.54_MA_0.41_Pb(I_0.98_Br_0.02_)_3_ with Os(atpy)(tbbpy)Cl^+^-rubrene-DBP doped PVA TTA-UC film with spiro-OMeTAD as a hole transport layer (Fig. 10a). TTA-UC integrated PVSC showed quadratic dependence of Δ*J*_sc_ with the 938 nm laser excitation arising due to the absorption of UC photons by the perovskite solar cell (Fig. 10a).^85^

They observed Δ*J*_sc_ = 0.1 mA cm^−2^ but at very high excitation intensity (4 W cm^−2^).

Further working in this direction, Tan, Chen, and co-workers, reported an enhancement of Δ*J*_sc_ = 2.38 mA cm^−2^ of MAPbI_3_ solar cell fabricated on rubrene-DBP layers at AM 1.5G illumination (Fig. 10b).^121^ Unlike the other TTA-UC integrated solar cell devices, they deposited TTA-UC layers between the electron transport layer (SnO_2_) and hole transport layer (Spiro-OMeTAD). Interestingly, they suggested the MAPbI_3_ layer acting both as a triplet sensitizer and solar cell. However, the exclusive contribution of TTA-UC to the total Δ*J*_sc_ enhancement is still disputed for this solar cell. It is because, the rubrene-DBP doping could have promoted perovskite growth owing to the formation of π-conjugated agglomerates, that served as suitable nuclei for better perovskite crystal packing. Hence, such a large enhancement in Δ*J*_sc_ can be partly attributed to better crystal packing of perovskite leading to enhanced Δ*J*_sc_.

**Fig. 11:**
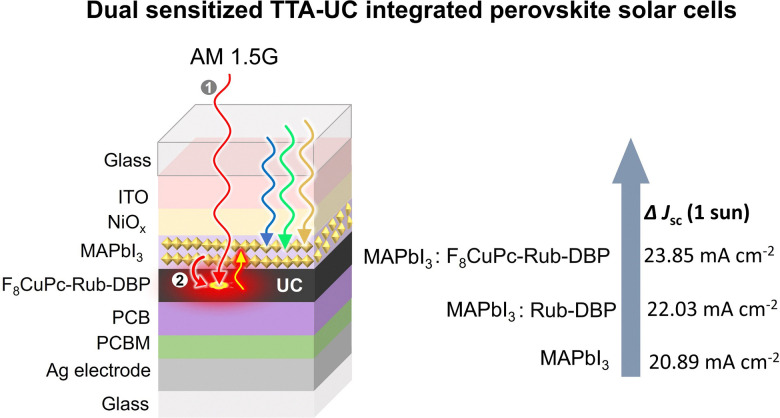
Illustration of the dual sensitized TTA-UC integrated perovskite solar cell and consequent Δ*J*_sc_ enhancement.

To shed doubts over the exclusive contribution of TTA-UC in Δ*J*_sc_ Tan, Chen, and co-workers reported a dual sensitization approach, where in addition to perovskite as sensitizer they doped the rubrene-DBP with NIR sensitizer, copper-2,3, 9,10,16,17,23,24-octa-fluoro phthalocyanine (F_8_CuPc). The resulting device (Fig. 11) showed enhancement of Δ*J*_sc_ = 2.96 mA cm^−2^ compared to the reference perovskite film and Δ*J*_sc_ = 1.14 mA cm^−2^ compared to the perovskite-rubrene-DBP film.^122^ Hence validating their claims. Additionally, the hydrophobic nature of F_8_CuPc acted as a moisture barrier against humidity.

While PVs can convert solar energy into electricity, storing electricity in PV modules for a long period has been an issue. Instead, the generated electricity can be stored in an electric battery bank. However, alternative technology, *i.e.* molecular solar thermal energy storage systems (MOST) can store solar energy in chemical bonds for a long period. The stored photon energy can be later released in the form of heat as per requirement. Hence, in the next section, we will discuss the potential of MOST and its implications with TTA-UC to increase the efficiency of storage.

## Photochemical energy storage driven by TTA-UC

**Fig. 12:**
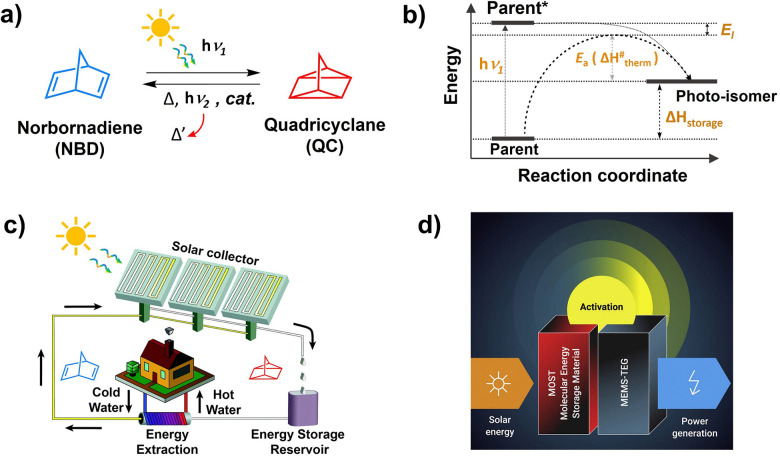
Illustration of (a) photothermal switching of norbornadiene–quadricyclane system, (b) energy diagram of photo-thermal switching, and application of MOST systems for (c) heating or cooling of homes and (d) electrical power generation. (b and c) is reproduced from ref. 17 with permission from the Royal Society of Chemistry. (d) is reproduced from ref. 18 with permission from Cell Press.

A typical MOST showing photo-thermal switching of Norbornadiene (NBD) ↔ Quadricyclane (QC) system is shown in Fig. 12a.^123^

Here the parent NBD undergoes photoisomerization to QC upon absorption of photon energy (*hv*_1_). The QC stores the photon energy in chemical bonds and releases it as thermal energy (Δ*H*_storage_) in a process of converting back to NBD activated by light (*hv*_2_), heat, or catalyst (Fig. 12a and b). The energy stored by photo-isomer can primarily be used for; (1) heating/cooling of houses in winter in temperate regions, and (2) electrical power generation upon integration with thermoelectric generators (Fig. 12c and d).^17,18^

**Fig. 13:**
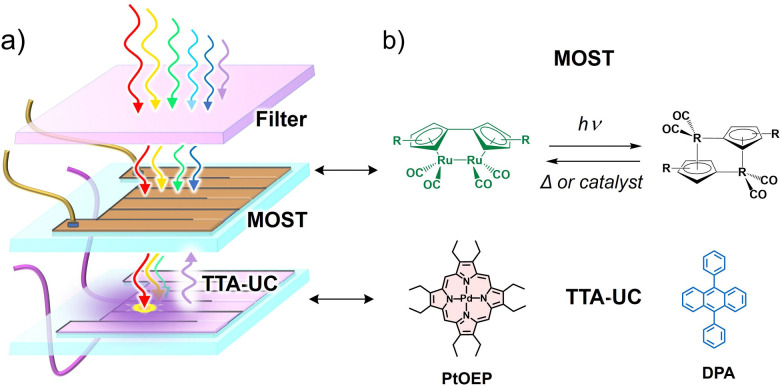
(a) Illustration of the microfluidic flow reactor comprising of TTA-UC fluidic chip integrated into molecular solar thermal energy storage fluidic chip (MOST). (b) Illustration of photothermal switching of fulvalene diruthenium derivative (green/black; R = 1,1-dimethyltridecyl) system (MOST), and molecular structures of the TTA-UC sensitizer (PdOEP) and annihilator (DPA).

Similar to PV devices, where solar cells have small bandgap limitations, the organic molecules used in MOST systems also have a small solar absorption window. Most of the organic molecules used in MOST systems absorb in the UVB-to-blue region which is a small part of the AM 1.5G solar spectrum and has a low spectral irradiance.^124^ Integration with TTA-UC materials can increase the solar flux due to the upconversion of a visible photon to UV photons (Fig. 13a). Hence, the Vis to UV TTA-UC materials are most relevant for MOST applications. However, not much work has been done in these directions due to many photophysical and device fabrication issues. Among the key issues is the overlap of the absorption/emission spectrum of the photo-switch with the sensitizer/annihilator pair. Therefore, the foremost challenge is to find suitable photoswitch/TTA-UC pair that does not interfere with each other. For example, in one such proof-of-concept demonstration, our research group integrated fulvalene diruthenium (FvRu_2_) derivative-based photothermal switch with palladium octa-ethyl porphyrin (PdOEP) and diphenyanthracene (DPA) based TTA-UC solution.^125^ The low energy FvRu_2_ has an absorption maximum at around 400 nm with a tail extending into the blue region that overlaps with the emission spectrum of DPA. Incidentally, the high-energy photo-isomers of FvRu_2_ do not absorb at all in the visible region.

**Fig. 14:**
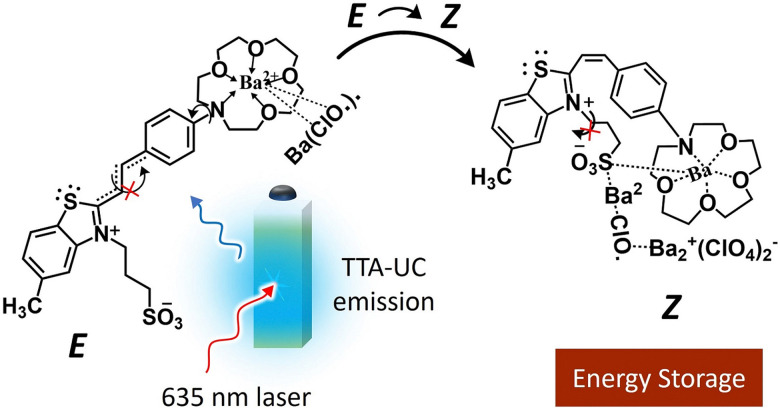
Illustration of the TTA-UC assisted *E* to *Z* isomerization of the barium complexed crown ether functionalized hemicyanine dyes upon 635 nm laser excitation.

Due to this optical transparency, the green photons could be upconverted to blue photons *via* TTA-UC using PdOEP as a sensitizer (Fig. 13b). Hence the conversion efficiency of FvRu_2_ could be increased by 130% due to the enhanced photon flux. Baluschev's research group has proposed the barium metal complexed *Z* isomers of crown ether functionalized hemicyanine dyes as a suitable candidate for photon energy storage upon *E* to *Z* photoisomerization with blue light excitation. To enhance the blue photon flux or shift the action spectrum toward a low energy, they integrated this system with a Perylene-PdTPBP-based TTA-UC solution that resulted in *E*–*Z* isomerization upon excitation with 635 nm laser (Fig. 14)^126^ However, they did not report back conversion of the *Z* isomer to release the stored photon energy. Such systems could become practically more relevant if designed in the solid-state (Fig. 12c and d). However, the small photon conversion quantum yields of these photos-witches are a limiting factor. Hence, photo-thermal switches with *ϕ*_C_ ∼ 100% could be a game changer.^123^ And specifically for TTA-UC integration there must be optical transparency for one of the photo isomer in the TTA-UC region.

## Summary and future perspectives

The progress in TTA-UC enhanced solar energy conversion systems is on a slow rise. The actual reason for the slow progress is the requirement of transparent high band gap solar cell devices, which themselves are at the early developmental stage. Therefore, research on finding an efficient solid-state broadband TTA-UC upconverter and corresponding suitable band gap solar cells are moving side by side. Therefore, the majority of the earliest work has been done by integrating liquid upconversion solution below the thin film solar cells (a-Si:H, P_3_HT:ICBA).^101–105^ Later annihilator adsorbed on TiO_2_ as an electron donor dye in DSSCs,^106–109,111,113–115^ and liquid/solid upconverter integrated conventional DSSCs were developed.^24,116^ Among these systems a maximum short circuit current density, Δ*J*_sc_ = 1.65 mA cm^−2^ has been recorded with solid-state photon upconverter (PdTPBP-perylene doped polyurethane film) upon 635 nm laser excitation equivalent to 1 sun.^116^ Well, it is greater than the threshold limit for PV's applications *i.e.* Δ*J*_sc_ = 0.1 mA cm^−2^.^112^ Hence, it highlighted the importance of solid-state photon upconverters for PV devices. Of late, perovskites solar cells have been integrated with TTA-UC upconverter either by placing TTA-UC film below the solar cell^85^ or fabricating them between hole and electron transport layers.^121,122^ The latter device design has yielded a maximum Δ*J*_sc_ enhancement of 2.96 mA cm^−2^ due to TTA-UC. Interestingly in addition to photon upconversion, TTA-UC layers assisted in better crystal packing of perovskites and improved moisture stability. This, show the importance of crystalline TTA-UC systems for PV integration to improve longevity. The performance could further be improved by co-crystallization of broad-band sensitizer–annihilator pairs for maximum harvesting of transmitted photons. Further development of efficient perovskite solar cells working above 1.62 eV in the visible region would exponentially increase their applications with efficient crystalline TTA-UC photon upconverters.^89,92,93^ But important consideration over here is the annihilator design with a high statistical probability factor in addition to triplet exciton diffusion length and lifetime in the crystalline state. We found that *f* is highly dependent on the emission wavelength and derivatization of the parent acene. However, in a suitable crystalline framework, it can be increased by recycling quintet and high-energy triplets *via* quintet–singlet spin mixing and reverse intersystem crossing.^60^

While PV technology is seen as a sustainable energy approach, commercial crystalline solar cells have a typical life of ≈25 years.^127^ In the past decade the cumulative PV capacity worldwide has boomed from 48 GW to 700 GW, which is also increasing the number of end-of-life (EOL) solar panels. Going by the current expansion of PV technology, the world will see ≈78 million tons of solar panel waste by 2050.^128^ In addition to the possible waste, the PV modules occupy a lot of space which adds to the cost. To address such issues, research on their recycling (down or upcycling) is already underway^129,130^ and alternative fabrication frameworks in the form of thin film plastic solar technologies are already in the market.^131^ But they may also face a similar fate regarding environmental waste since they are generally fabricated on polyester or polyimide sheets which are synthetic plastic.^132^ Therefore, replacing the fabrication material with robust biomaterial can enhance the sustainability factor since it could degrade in the natural environment after recycling chemicals at the EOL. We have demonstrated such a possibility by fabricating efficient broadband solid-state TTA-UC bioplastics using protein as an encapsulator to synthetic chromophores and their consequent recycling at the EOL.^31,32^ Such biopolymers can also be used to fabricate solar cell materials to replace polyester or polyimide sheets. For example, most earth-abundant biopolymers like cellulose and chitosan possess suitable dielectric and mechanical properties as that of polyester required for solar cells.^133–135^ Moreover, coating these biopolymers with Nylon 11 which is a natural polyimide derived from castor beans can decrease the water vapor permeability. For example, recently Gao *et al.* used flexible nanocellulose coated with acrylic resin as a back-sheet substrate for the fabrication of perovskite solar cells that showed a power conversion efficiency of 4.25%.^136^ We believe that the fabrication of more such bio-fabricated solar cells and further integration with TTA-UC bioplastics would pave the way for future bioplastic solar cells that our research group is committed to achieving.

Regarding TTA-UC integrated MOST systems, there has not been great progress due to many spectral factors related to both photo-switch and TTA-UC systems. Over the years our research group has developed various MOST devices with several NBD, azobenzene, and dihydroazulene derivatives-based photo-switches to harvest maximum solar photons.^123,137^ The NBD ↔ QC system has a high *ϕ*_c_ in addition to the transparent visible spectral window beyond 400 nm that could be utilized for integration with an efficient Vis to UV TTA-UC system. Because NBD and its derivatives typically absorbs between 320 to 450 nm, its photo-isomerization yield can be increase by increasing solar flux in this region by upconverting 400–550 nm light using suitable sensitizer–annihilator pair. However, low UC efficiency of Vis to UV systems over the years has been a big obstacle that has now been sorted with the discovery of highly efficient TIPS-naphthalene based TTA-UC systems near the sub-solar irradiance.^36,79^ The TIPS-naphthalene emits between 300–400 nm which is in the absorption range of NBD. Hence are suitable for efficiency enhancement of the NBD ↔ QC systems in ambient conditions. However, such systems need to be developed in the solid-state for practical fabrication with the solid-state MOST devices, which our research group is committed to achieving.

## Author contributions

Lukas Naimovičius, Pankaj Bharmoria, and Kasper Moth-Poulsen conceptualized the idea, wrote the first draft, revised, and edited the final draft of this manuscript.

## Conflicts of interest

There are no conflicts to declare.

## Supplementary Material

## References

[cit1] Blankenship R. E. (2010). Early Evolution of Photosynthesis. Plant Physiol..

[cit2] Hannibal M. E. , The history and future of fire, 2021, vol. 374

[cit3] Buchanan M. (2017). Energy transitions. Nat. Phys..

[cit4] Kerr R. A. , Humans Fueled Global Warming Millennia Ago, 2013, vol. 342 10.1126/science.342.6161.918 24264968

[cit5] Austin G. and Sugihara K., Labour-Intensive Industrialization in Global History, Taylor & Francis, 2013

[cit6] Paris Agreement, United Nations Treaty Collect., 2016, XXVII.7.d

[cit7] Green M., Dunlop E., Hohl-Ebinger J., Yoshita M., Kopidakis N., Hao X. (2021). Solar cell efficiency tables (version 57). Prog. Photovoltaics Res. Appl..

[cit8] Shockley W., Queisser H. J. (1961). Detailed balance limit of efficiency of p–n junction solar cells. J. Appl. Phys..

[cit9] Shah A., Torres P., Tscharner R., Wyrsch N., Keppner H. (1999). Photovoltaic technology: the case for thin-film solar cells. Science.

[cit10] McGehee M. D. (2011). Paradigm shifts in dye-sensitized solar cells. Science.

[cit11] Muñoz-García A. B., Benesperi I., Boschloo G., Concepcion J. J., Delcamp J. H., Gibson E. A., Meyer G. J., Pavone M., Pettersson H., Hagfeldt A., Freitag M. (2021). Dye-sensitized solar cells strike back. Chem. Soc. Rev..

[cit12] Dimroth F., Tibbits T. N. D., Niemeyer M., Predan F., Beutel P., Karcher C., Oliva E., Siefer G., Lackner D., Fuß-Kailuweit P., Bett A. W., Krause R., Drazek C., Guiot E., Wasselin J., Tauzin A., Signamarcheix T. (2016). Four-Junction Wafer-Bonded Concentrator Solar Cells. IEEE J. Photovoltaics.

[cit13] Juarez-Perez E. J., Haro M. (2020). Perovskite solar cells take a step forward. Science.

[cit14] Ghazy A., Safdar M., Lastusaari M., Savin H., Karppinen M. (2021). Advances in upconversion enhanced solar cell performance. Sol. Energy Mater. Sol. Cells.

[cit15] Carrod A. J., Gray V., Börjesson K. (2022). Recent advances in triplet–triplet annihilation upconversion and singlet fission, towards solar energy applications. Energy Environ. Sci..

[cit16] Pedrini J., Monguzzi A. (2017). Recent advances in the application triplet–triplet annihilation-based photon upconversion systems to solar technologies. J. Photonics Energy.

[cit17] Wang Z., Roffey A., Losantos R., Lennartson A., Jevric M., Petersen A. U., Quant M., Dreos A., Wen X., Sampedro D., Börjesson K., Moth-Poulsen K. (2019). Macroscopic heat release in a molecular solar thermal energy storage system. Energy Environ. Sci..

[cit18] Wang Z., Wu Z., Hu Z., Hernández J. O., Mu E., Zhang Z. Y., Jevric M., Liu Y., Fu X., Wang F., Li T., Moth-Poulsen K. (2022). Chip-scale solar thermal electrical power generation. Cell Rep. Phys. Sci..

[cit19] Parker C. A., Hatchard C. G. (1962). Delayed fluorescence from solutions of anthracene and phenanthrene. Proc. R. Soc. London, Ser. A.

[cit20] Keivanidis P. E., Baluschev S., Miteva T., Nelles G., Scherf U., Yasuda A., Wegner G. (2003). Up-Conversion Photoluminescence in Polyfluorene Doped with Metal(ii)-Octaethyl Porphyrins. Adv. Mater..

[cit21] Bharmoria P., Bildirir H., Moth-Poulsen K. (2020). Triplet–triplet annihilation based near infrared to visible molecular photon upconversion. Chem. Soc. Rev..

[cit22] Gray V., Moth-Poulsen K., Albinsson B., Abrahamsson M. (2018). Towards efficient solid-state triplet–triplet annihilation based photon upconversion: supramolecular, macromolecular and self-assembled systems. Coord. Chem. Rev..

[cit23] Sasaki Y., Amemori S., Yanai N., Kimizuka N. (2021). Singlet-to-Triplet Absorption for Near-Infrared-to-Visible Photon Upconversion. Bull. Chem. Soc. Jpn..

[cit24] Nattestad A., Cheng Y. Y., Macqueen R. W., Schulze T. F., Thompson F. W., Mozer A. J., Fückel B., Khoury T., Crossley M. J., Lips K., Wallace G. G., Schmidt T. W. (2013). Dye-sensitized solar cell with integrated triplet–triplet annihilation upconversion system. J. Phys. Chem. Lett..

[cit25] Baluschev S., Yakutkin V., Miteva T., Avlasevich Y., Chernov S., Aleshchenkov S., Nelles G., Cheprakov A., Yasuda A., Müllen K., Wegner G. (2007). Blue-green up-conversion: noncoherent excitation by NIR light. Angew. Chem., Int. Ed..

[cit26] Zhou Y., Castellano F. N., Schmidt T. W., Hanson K. (2020). On the Quantum Yield of Photon Upconversion via Triplet–Triplet Annihilation. ACS Energy Lett..

[cit27] Dexter D. L. (1953). A theory of sensitized luminescence in solids. J. Chem. Phys..

[cit28] Resch-Genger U., Rurack K. (2013). Determination of the photoluminescence quantum yield of dilute dye solutions (IUPAC Technical Report). Pure Appl. Chem..

[cit29] Bharmoria P., Hisamitsu S., Nagatomi H., Ogawa T., Morikawa M. A., Yanai N., Kimizuka N. (2018). Simple and Versatile Platform for Air-Tolerant Photon Upconverting Hydrogels by Biopolymer-Surfactant-Chromophore Co-assembly. J. Am. Chem. Soc..

[cit30] Yanai N., Suzuki K., Ogawa T., Sasaki Y., Harada N., Kimizuka N. (2019). Absolute Method to Certify Quantum Yields of Photon Upconversion via Triplet–Triplet Annihilation. J. Phys. Chem. A.

[cit31] Bharmoria P., Hisamitsu S., Sasaki Y., Kang T. S., Morikawa M. A., Joarder B., Moth-Poulsen K., Bildirir H., Mårtensson A., Yanai N., Kimizuka N. (2021). Photon upconverting bioplastics with high efficiency and in-air durability. J. Mater. Chem. C.

[cit32] Bharmoria P., Edhborg F., Bildirir H., Sasaki Y., Ghasemi S., Mårtensson A., Yanai N., Kimizuka N., Albinsson B., Börjesson K., Moth-Poulsen K. (2022). Recyclable optical bioplastics platform for solid state red light harvesting via triplet–triplet annihilation photon upconversion. J. Mater. Chem. A.

[cit33] Brouwer A. M. (2011). Standards for photoluminescence quantum yield measurements in solution (IUPAC technical report). Pure Appl. Chem..

[cit34] Josefsen L. B., Boyle R. W. (2008). Photodynamic Therapy and the Development of Metal-Based Photosensitisers. Met.-Based Drugs.

[cit35] Mahmood Z. , Ji S., Zhao J., Hussain M., Sadiq F., Rehmat N. and Imran M., in Emerging Strategies to Reduce Transmission and Thermalization Losses in Solar Cells: Redefining the Limits of Solar Power Conversion Efficiency, ed. J. S. Lissau and M. Madsen, Springer International Publishing, Cham, 2022, pp. 71–105

[cit36] Olesund A., Johnsson J., Edhborg F., Ghasemi S., Moth-Poulsen K., Albinsson B. (2022). Approaching the Spin-Statistical Limit in Visible-to-Ultraviolet Photon Upconversion. J. Am. Chem. Soc..

[cit37] Radiunas E., Raišys S., Juršenas S., Jozeliunaite A., Javorskis T., Šinkeviciute U., Orentas E., Kazlauskas K. (2020). Understanding the limitations of NIR-to-visible photon upconversion in phthalocyanine-sensitized rubrene systems. J. Mater. Chem. C.

[cit38] Haruki R., Sasaki Y., Masutani K., Yanai N., Kimizuka N. (2020). Leaping across the visible range: near-infrared-to-violet photon upconversion employing a silyl-substituted anthracene. Chem. Commun..

[cit39] Yanai N., Kimizuka N. (2017). New Triplet Sensitization Routes for Photon Upconversion: Thermally Activated Delayed Fluorescence Molecules, Inorganic Nanocrystals, and Singlet-to-Triplet Absorption. Acc. Chem. Res..

[cit40] Wei Y., Pan K., Cao X., Li Y., Zhou X., Yang C. (2022). Multiple Resonance Thermally Activated Delayed Fluorescence Sensitizers Enable Green-to-Ultraviolet Photon Upconversion: Application in Photochemical Transformations. CCS Chem..

[cit41] Bassan E., Gualandi A., Cozzi P. G., Ceroni P. (2021). Design of BODIPY dyes as triplet photosensitizers: electronic properties tailored for solar energy conversion, photoredox catalysis and photodynamic therapy. Chem. Sci..

[cit42] Lin X., Chen Z., Han Y., Nie C., Xia P., He S., Li J., Wu K. (2022). ZnSe/ZnS Core/Shell Quantum Dots as Triplet Sensitizers toward Visible-to-Ultraviolet B Photon Upconversion. ACS Energy Lett..

[cit43] Wu M., Congreve D. N., Wilson M. W. B., Jean J., Geva N., Welborn M., Van Voorhis T., Bulovic V., Bawendi M. G., Baldo M. A. (2016). Solid-state infrared-to-visible upconversion sensitized by colloidal nanocrystals. Nat. Photonics.

[cit44] Mahboub M., Huang Z., Tang M. L. (2016). Efficient Infrared-to-Visible Upconversion with Subsolar Irradiance. Nano Lett..

[cit45] Vanorman Z. A., Nienhaus L. (2021). Bulk Metal Halide Perovskites as Triplet Sensitizers: Taking Charge of Upconversion. ACS Energy Lett..

[cit46] Carrod A. J., Cravcenco A., Ye C., Börjesson K. (2022). Modulating TTA efficiency through control of high energy triplet states. J. Mater. Chem. C.

[cit47] Radiunas E., Naimovičius L., Raišys S., Jozeliūnaitė A., Orentas E., Kazlauskas K. (2022). Efficient NIR-to-vis photon upconversion in binary rubrene films deposited by simplified thermal evaporation. J. Mater. Chem. C.

[cit48] Edhborg F., Bildirir H., Bharmoria P., Moth-Poulsen K., Albinsson B. (2021). Intramolecular Triplet–Triplet Annihilation Photon Upconversion in Diffusionally Restricted Anthracene Polymer. J. Phys. Chem. B.

[cit49] Olesund A., Gray V., Mårtensson J., Albinsson B. (2021). Diphenylanthracene Dimers for Triplet–Triplet Annihilation Photon Upconversion: Mechanistic Insights for Intramolecular Pathways and the Importance of Molecular Geometry. J. Am. Chem. Soc..

[cit50] Congrave D. G., Drummond B. H., Gray V., Bond A. D., Rao A., Friend R. H., Bronstein H. (2021). Suppressing aggregation induced quenching in anthracene based conjugated polymers. Polym. Chem..

[cit51] Rigsby E. M., Miyashita T., Fishman D. A., Roberts S. T., Tang M. L. (2021). CdSe nanocrystal sensitized photon upconverting film. RSC Adv..

[cit52] Durandin N. A., Isokuortti J., Efimov A., Vuorimaa-Laukkanen E., Tkachenko N. V., Laaksonen T. (2019). Critical Sensitizer Quality Attributes for Efficient Triplet–Triplet Annihilation Upconversion with Low Power Density Thresholds. J. Phys. Chem. C.

[cit53] Murakami Y., Kamada K. (2021). Kinetics of photon upconversion by triplet–triplet annihilation: a comprehensive tutorial. Phys. Chem. Chem. Phys..

[cit54] Monguzzi A., Mézyk J., Scotognella F., Tubino R., Meinardi F. (2008). Upconversion-induced fluorescence in multicomponent systems: steady-state excitation power threshold. Phys. Rev. B: Condens. Matter Mater. Phys..

[cit55] Jacobs S. J., Shultz D. A., Jain R., Novak J., Dougherty D. A. (1993). Evaluation of potential ferromagnetic coupling units: the bis(TMM) [bis(trimethylenemethane)] approach to high-spin organic molecules. J. Am. Chem. Soc..

[cit56] Itoh K. (1978). Electronic structures of aromatic hydrocarbons with high spin multiplicities in the electronic ground state. Pure Appl. Chem..

[cit57] Köhler A. and Bassler H., Bimolecular processes, Electronic processes inorganic semiconductors, Wiley-VCH, 2015, pp. 287–292

[cit58] Wang X., Tom R., Liu X., Congreve D. N., Marom N. (2020). An energetics perspective on why there are so few triplet–triplet annihilation emitters. J. Mater. Chem. C.

[cit59] Luo Y., Zhang K., Ding Z., Chen P., Peng X., Zhao Y., Chen K., Li C., Zheng X., Huang Y., Pu X., Liu Y., Su S.-J., Hou X., Lu Z. (2022). Ultra-fast triplet–triplet-annihilation-mediated high-lying reverse intersystem crossing triggered by participation of nπ*-featured excited states. Nat. Commun..

[cit60] Ha D.-G., Wan R., Kim C. A., Lin T.-A., Yang L., Van Voorhis T., Baldo M. A., Dincă M. (2022). Exchange controlled triplet fusion in metal–organic frameworks. Nat. Mater..

[cit61] Naimovičius L., Radiunas E., Chatinovska B., Jozeliūnaitė A., Orentas E., Kazlauskas K. (2023). Functionalized diketopyrrolopyrrole compounds for NIR-to-visible photon upconversion. J. Mater. Chem. C.

[cit62] Beljonne D., Rao A. (2019). Photon Upconversion from Near-Infrared to Blue Light with TIPS-Anthracene as an E ffi cient Triplet−Triplet Annihilator. ACS Mater. Lett..

[cit63] Monguzzi A., Tubino R., Hoseinkhani S., Campione M., Meinardi F. (2012). Low power, non-coherent sensitized photon up-conversion: modelling and perspectives. Phys. Chem. Chem. Phys..

[cit64] Wang X., Marom N. (2022). An energetics assessment of benzo[*a*]tetracene and benzo[*a*]pyrene as triplet–triplet annihilation emitters. Mol. Syst. Des. Eng..

[cit65] Raišys S., Juršėnas S., Kazlauskas K. (2022). Boost in Solid-State Photon Upconversion Efficiency through Combined Approach of Melt-Processing and Purification. Sol. RRL.

[cit66] Gray V., Dreos A., Erhart P., Albinsson B., Moth-poulsen K., Abrahamsson M. (2017). Loss channels in triplet – triplet annihilation photon upconversion: importance of annihilator singlet and triplet surface shapes. Phys. Chem. Chem. Phys..

[cit67] Zhou Q., Zhou M., Wei Y., Zhou X., Liu S., Zhang S., Zhang B. (2017). Solvent effects on the triplet–triplet annihilation upconversion of diiodo-Bodipy and perylene. Phys. Chem. Chem. Phys..

[cit68] Hoseinkhani S., Tubino R., Meinardi F., Monguzzi A. (2015). Achieving the photon up-conversion thermodynamic yield upper limit by sensitized triplet–triplet annihilation. Phys. Chem. Chem. Phys..

[cit69] Groff R. P., Merrifield R. E., Avakian P., Station E. (1970). Singlet and triplet channels for triplet-exciton fusion in anthracene crystals. Chem. Phys. Lett..

[cit70] Fourny J., Delacôte G. (1970). Comparison of high and zero magnetic field values of non radiative triplet–triplet annihilation rate constants in crystalline anthracene. Chem. Phys. Lett..

[cit71] Ern V., Saint-Clair J. L., Schott M., Delacote G. (1971). Effects of exciton interactions on the fluorescence yield of crystalline tetracene. Chem. Phys. Lett..

[cit72] Bossanyi D. G., Sasaki Y., Wang S., Chekulaev D., Kimizuka N., Yanai N., Clark J. (2021). Spin Statistics for Triplet–Triplet Annihilation Upconversion: Exchange Coupling, Intermolecular Orientation, and Reverse Intersystem Crossing. JACS Au.

[cit73] Cheng Y. Y., Fuckel B., Khoury T., Clady R. G. C. R., Tayebjee M. J. Y., Ekins-Daukes N. J., Crossley M. J., Schmidt T. W. (2010). Kinetic Analysis of Photochemical Upconversion by Triplet–Triplet Annihilation: Beyond Any Spin Statistical Limit. J. Phys. Chem. Lett..

[cit74] Baronas P., Kreiza G., Naimovičius L., Radiunas E., Kazlauskas K., Orentas E., Juršėnas S. (2022). Sweet Spot of Intermolecular Coupling in Crystalline Rubrene: Intermolecular Separation to Minimize Singlet Fission and Retain Triplet–Triplet Annihilation. J. Phys. Chem. C.

[cit75] Englman R., Jortner J. (1970). The energy gap law for radiationless transitions in large molecules. Mol. Phys..

[cit76] Siebrand W., Transitions Radiationless (1967). in Polyatomic Molecules. II. Triplet-Ground-State Transitions in Aromatic Hydrocarbons. J. Chem. Phys..

[cit77] Sun W., Ronchi A., Zhao T., Han J., Monguzzi A., Duan P. (2021). Highly efficient photon upconversion based on triplet–triplet annihilation from bichromophoric annihilators. J. Mater. Chem. C.

[cit78] Wei Y., Li Y., Li Z., Xu X., Cao X., Zhou X., Yang C. (2021). Efficient Triplet–Triplet Annihilation Upconversion in Solution and Hydrogel Enabled by an S–T Absorption Os(ii) Complex Dyad with an Elongated Triplet Lifetime. Inorg. Chem..

[cit79] Harada N., Sasaki Y., Hosoyamada M., Kimizuka N., Yanai N. (2021). Discovery of Key TIPS-Naphthalene for Efficient Visible-to-UV Photon Upconversion under Sunlight and Room Light. Angew. Chem., Int. Ed..

[cit80] Joarder B., Yanai N., Kimizuka N. (2018). Solid-State Photon Upconversion Materials: Structural Integrity and Triplet-Singlet Dual Energy Migration. J. Phys. Chem. Lett..

[cit81] Alves J., Feng J., Nienhaus L., Schmidt T. W. (2022). Challenges, progress and prospects in solid state triplet fusion upconversion. J. Mater. Chem. C.

[cit82] Abulikemu A., Sakagami Y., Heck C., Kamada K., Sotome H., Miyasaka H., Kuzuhara D., Yamada H. (2019). Solid-State, Near-Infrared to Visible Photon Upconversion via Triplet–Triplet
Annihilation of a Binary System Fabricated by Solution Casting. ACS Appl. Mater. Interfaces.

[cit83] Radiunas E., Dapkevičius M., Raišys S., Juršenas S., Jozeliunaite A., Javorskis T., Šinkevičiute U., Orentas E., Kazlauskas K. (2020). Impact of *T*-butyl substitution in a rubrene emitter for solid state NIR-to-visible photon upconversion. Phys. Chem. Chem. Phys..

[cit84] Lin T. A., Perkinson C. F., Baldo M. A. (2020). Strategies for High-Performance Solid-State Triplet–Triplet-Annihilation-Based Photon Upconversion. Adv. Mater..

[cit85] Kinoshita M., Sasaki Y., Amemori S., Harada N., Hu Z., Liu Z., Ono L. K., Qi Y., Yanai N., Kimizuka N. (2020). Photon Upconverting Solid Films with Improved Efficiency for Endowing Perovskite Solar Cells with Near-Infrared Sensitivity. ChemPhotoChem.

[cit86] Nienhaus L., Wu M., Geva N., Shepherd J. J., Wilson M. W. B., Bulović V., Van Voorhis T., Baldo M. A., Bawendi M. G. (2017). Speed Limit for Triplet-Exciton Transfer in Solid-State PbS Nanocrystal-Sensitized Photon Upconversion. ACS Nano.

[cit87] Duan J., Liu Y., Zhang Y., Chen Z., Xu X., Ye L., Wang Z., Yang Y., Zhang D., Zhu H. (2022). Efficient solid-state infrared-to-visible photon upconversion on atomically thin monolayer semiconductors. Sci. Adv..

[cit88] Takeshi Mori S. M. , Mori T., Saito A., Masuda T., Saomoto H. and Hagihara M., High-Efficiency Near-Infrared-to-Visible Photon Upconversion in Poly(vinyl alcohol) Porous Film, *ChemRxiv*, 2022, preprint 10.26434/chemrxiv-2022-mjjxb-v2 37015037

[cit89] Li L., Zeng Y., Chen J., Yu T., Hu R., Yang G., Li Y. (2019). Thermally Activated Delayed Fluorescence via Triplet Fusion. J. Phys. Chem. Lett..

[cit90] Vadrucci R., Monguzzi A., Saenz F., Wilts B. D., Simon Y. C., Weder C. (2017). Nanodroplet-Containing Polymers for Efficient Low-Power Light Upconversion. Adv. Mater..

[cit91] Kim J., Deng F., Castellano F. N., Kim J. (2012). High Efficiency Low-Power Upconverting Soft Materials. Chem. Mater..

[cit92] Enomoto R., Hoshi M., Oyama H., Agata H., Kurokawa S., Kuma H., Uekusa H., Murakami Y. (2021). van der Waals solid solution crystals for highly efficient in-air photon upconversion under subsolar irradiance. Mater. Horiz..

[cit93] Kamada K., Sakagami Y., Mizokuro T., Fujiwara Y., Kobayashi K., Narushima K., Hirata S., Vacha M. (2017). Efficient triplet–triplet annihilation upconversion in binary crystalline solids fabricated: Via solution casting and operated in air. Mater. Horiz..

[cit94] Battaglia C., Cuevas A., De S. (2016). Wolf, High-efficiency crystalline silicon solar cells: status and perspectives. Energy Environ. Sci..

[cit95] Börjesson K., Lennartson A., Moth-Poulsen K. (2013). Efficiency Limit of Molecular Solar Thermal Energy Collecting Devices. ACS Sustainable Chem. Eng..

[cit96] Bharmoria P., Ghasemi S., Edhborg F., Losantos R., Wang Z., Mårtensson A., Morikawa M., Kimizuka N., İşci Ü., Dumoulin F., Albinsson B., Moth-Poulsen K. (2022). Far-red triplet sensitized Z-to-E photoswitching of azobenzene in bioplastics. Chem. Sci..

[cit97] Bren V. A., Dubonosov A. D., Minkin V. I., Chernoivanov V. A. (1991). Norbornadiene–quadricyclane—an effective molecular system for the storage of solar energy. Russ. Chem. Rev..

[cit98] Lennartson A., Roffey A., Moth-Poulsen K. (2015). Designing photoswitches for molecular solar thermal energy storage. Tetrahedron Lett..

[cit99] Zähringer T. J. B., Moghtader J. A., Bertrams M., Roy B., Uji M., Yanai N., Kerzig C. (2022). Blue-to-UVB Upconversion, Solvent Sensitization and Challenging Bond Activation Enabled by a Benzene-Based Annihilator. Angew. Chem., Int. Ed..

[cit100] Jiang X., Guo X., Peng J., Zhao D. (2016). Triplet − Triplet Annihilation Photon Upconversion in Polymer Thin Film: Sensitizer Design. ACS Appl. Mater. Interfaces.

[cit101] Cheng Y. Y., Fückel B., MacQueen R. W., Khoury T., Clady R. G. C. R., Schulze T. F., Ekins-Daukes N. J., Crossley M. J., Stannowski B., Lips K., Schmidt T. W. (2012). Improving the light-harvesting of amorphous silicon solar cells with photochemical upconversion. Energy Environ. Sci..

[cit102] Schulze T. F., Cheng Y. Y., Fückel B., MacQueen R. W., Danos A., Davis N. J. L. K., Tayebjee M. J. Y., Khoury T., Clady R. G. C. R., Ekins-Daukes N. J., Crossley M. J., Stannowski B., Lips K., Schmidt T. W. (2012). Photochemical Upconversion Enhanced Solar Cells: Effect of a Back Reflector. Aust. J. Chem..

[cit103] Schulze T. F., Czolk J., Cheng Y. Y., Fückel B., MacQueen R. W., Khoury T., Crossley M. J., Stannowski B., Lips K., Lemmer U., Colsmann A., Schmidt T. W. (2012). Efficiency enhancement of organic and thin-film silicon solar cells with photochemical upconversion. J. Phys. Chem. C.

[cit104] Schulze T. F., Cheng Y. Y., Khoury T. H., Crossley M., Stannowski B., Lips K., Schmidt T. W. (2013). Micro-optical design of photochemical upconverters for thin-film solar cells. J. Photonics Energy.

[cit105] Cheng Y. Y., Nattestad A., Schulze T. F., Macqueen R. W., Fückel B., Lips K., Wallace G. G., Khoury T., Crossley M. J., Schmidt T. W. (2016). Increased upconversion performance for thin film solar cells: a trimolecular composition. Chem. Sci..

[cit106] Simpson C., Clarke T. M., MacQueen R. W., Cheng Y. Y., Trevitt A. J., Mozer A. J., Wagner P., Schmidt T. W., Nattestad A. (2015). An intermediate band dye-sensitised solar cell using triplet–triplet annihilation. Phys. Chem. Chem. Phys..

[cit107] Hill S. P., Banerjee T., Dilbeck T., Hanson K. (2015). Photon Upconversion and Photocurrent Generation via Self-Assembly at Organic-Inorganic Interfaces. J. Phys. Chem. Lett..

[cit108] Hill S. P., Dilbeck T., Baduell E., Hanson K. (2016). Integrated Photon Upconversion Solar Cell via Molecular Self-Assembled Bilayers. ACS Energy Lett..

[cit109] Dilbeck T., Hill S. P., Hanson K. (2017). Harnessing molecular photon upconversion at sub-solar irradiance using dual sensitized self-assembled trilayers. J. Mater. Chem. A.

[cit110] Beery D., Arcidiacono A., Wheeler J. P., Chen J., Hanson K. (2022). Harnessing near-infrared light via S0 to T1 sensitizer excitation in a molecular photon upconversion solar cell. J. Mater. Chem. C.

[cit111] Morifuji T., Takekuma Y., Nagata M. (2019). Integrated Photon Upconversion Dye-Sensitized Solar Cell by Co-adsorption with Derivative of Pt-Porphyrin and Anthracene on Mesoporous TiO_2_. ACS Omega.

[cit112] Frazer L., Gallaher J. K., Schmidt T. W. (2017). Optimizing the Efficiency of Solar Photon Upconversion. ACS Energy Lett..

[cit113] Bharmoria P. and Ventura S. P. M., in Nanomaterials for Healthcare, Energy and Environment, ed. A. H. Bhat, I. Khan, M. Jawaid, F. O. Suliman, H. Al-Lawati and S. M. Al-Kindy, Springer Singapore, Singapore, 2019, pp. 1–29

[cit114] Beery D., Wheeler J. P., Arcidiacono A., Hanson K. (2020). CdSe Quantum Dot Sensitized Molecular Photon Upconversion Solar Cells. ACS Appl. Energy Mater..

[cit115] Zhou Y., Ruchlin C., Robb A. J., Hanson K. (2019). Singlet Sensitization-Enhanced Upconversion Solar Cells via Self-Assembled Trilayers. ACS Energy Lett..

[cit116] Li C., Koenigsmann C., Deng F., Hagstrom A., Schmuttenmaer C. A., Kim J. H. (2016). Photocurrent Enhancement from Solid-State Triplet–Triplet Annihilation Upconversion of Low-Intensity, Low-Energy Photons. ACS Photonics.

[cit117] Kim J. Y., Lee J. W., Jung H. S., Shin H., Park N. G. (2020). High-Efficiency Perovskite Solar Cells. Chem. Rev..

[cit118] Sarritzu V., Sestu N., Marongiu D., Chang X., Wang Q., Masi S., Colella S., Rizzo A., Gocalinska A., Pelucchi E., Mercuri M. L., Quochi F., Saba M., Mura A., Bongiovanni G. (2018). Direct or Indirect Bandgap in Hybrid Lead Halide Perovskites?. Adv. Opt. Mater..

[cit119] National Renewable Energy Laboratory (NREL), Best Research-Cell Efficiencies

[cit120] Gholizadeh E. M., Prasad S. K. K., Teh Z. L., Ishwara T., Norman S., Petty A. J., Cole J. H., Cheong S., Tilley R. D., Anthony J. E., Huang S., Schmidt T. W. (2020). Photochemical upconversion of near-infrared light from below the silicon bandgap. Nat. Photonics.

[cit121] Sheng W., Yang J., Li X., Liu G., Lin Z., Long J., Xiao S., Tan L., Chen Y. (2021). Tremendously enhanced photocurrent enabled by triplet–triplet annihilation up-conversion for high-performance perovskite solar cells. Energy Environ. Sci..

[cit122] Sheng W., Yang J., Li X., Zhang J., Su Y., Zhong Y., Zhang Y., Gong L., Tan L., Chen Y. (2022). Dual Triplet Sensitization Strategy for Efficient and Stable Triplet–Triplet Annihilation Upconversion Perovskite Solar Cells. CCS Chem..

[cit123] Wang Z., Hölzel H., Moth-Poulsen K. (2022). Status and challenges for molecular solar thermal energy storage system based devices. Chem. Soc. Rev..

[cit124] Kasten F., Young A. T. (1989). Revised optical air mass tables and approximation formula. Appl. Opt..

[cit125] Börjesson K., Dzebo D., Albinsson B., Moth-Poulsen K. (2013). Photon upconversion facilitated molecular solar energy storage. J. Mater. Chem. A.

[cit126] Vasilev A., Dimitrova R., Kandinska M., Landfester K., Baluschev S. (2021). Accumulation of the photonic energy of the deep-red part of the terrestrial sun irradiation by rare-earth metal-free E–Z photoisomerization. J. Mater. Chem. C.

[cit127] Chowdhury M. S., Rahman K. S., Chowdhury T., Nuthammachot N., Techato K., Akhtaruzzaman M., Tiong S. K., Sopian K., Amin N. (2020). An overview of solar photovoltaic panels’ end-of-life material recycling. Energy Strateg. Rev..

[cit128] Wang X., Tian X., Chen X., Ren L., Geng C. (2022). A review of end-of-life crystalline silicon solar photovoltaic panel recycling technology. Sol. Energy Mater. Sol. Cells.

[cit129] Xu Y., Li J., Tan Q., Peters A. L., Yang C. (2018). Global status of recycling waste solar panels: a review. Waste Manage..

[cit130] Tao M., Fthenakis V., Ebin B., Steenari B.-M., Butler E., Sinha P., Corkish R., Wambach K., Simon E. S. (2020). Major challenges and opportunities in silicon solar module recycling. Prog. Photovoltaics Res. Appl..

[cit131] Service R. F. (2011). Science.

[cit132] Rath J. K., Brinza M., Liu Y., Borreman A., Schropp R. E. I. (2010). Fabrication of thin film silicon solar cells on plastic substrate by very high frequency PECVD. Sol. Energy Mater. Sol. Cells.

[cit133] Petritz A., Wolfberger A., Fian A., Irimia-Vladu M., Haase A., Gold H., Rothländer T., Griesser T., Stadlober B. (2013). Cellulose as biodegradable high-k dielectric layer in organic complementary inverters. Appl. Phys. Lett..

[cit134] Nainggolan I., Nasution T. I., Putri S. R. E., Azdena D., Balyan M., Agusnar H. (2018). Study on chitosan film properties as a green dielectric. IOP Conf.: Ser. Mater. Sci. Eng..

[cit135] Lima C. G. A., de Oliveira R. S., Figueiró S. D., Wehmann C. F., Góes J. C., Sombra A. S. B. (2006). DC conductivity and dielectric permittivity of collagen–chitosan films. Mater. Chem. Phys..

[cit136] Gao L., Chao L., Hou M., Liang J., Chen Y., Yu H.-D., Huang W. (2019). Flexible, transparent nanocellulose paper-based perovskite solar cells. npj Flex. Electron..

[cit137] Wang Z., Moïse H., Cacciarini M., Nielsen M. B., Morikawa M., Kimizuka N., Moth-Poulsen K. (2021). Liquid-Based Multijunction Molecular Solar Thermal Energy Collection Device. Adv. Sci..

[cit138] Izawa S., Hiramoto M. (2021). Efficient solid-state photon upconversion enabled by triplet formation at an organic semiconductor interface. Nat. Photonics.

[cit139] Wei L., Fan C., Rao M., Gao F., He C., Sun Y., Zhu S., He Q., Yang C., Wu W. (2022). Triplet–triplet annihilation upconversion in LAPONITE®/PVP nanocomposites: absolute quantum yields of up to 23.8% in the solid state and application to anti-counterfeiting. Mater. Horiz..

